# Supplementary material for: The flavonoid 4,4′-dimethoxychalcone promotes autophagy-dependent longevity across species
Source: Nat Commun. 2019 Feb 19;10:651. doi: 10.1038/s41467-019-08555-w (PMC6381180; doi:10.1038/s41467-019-08555-w)
Supplement: Supplementary file 1 — Supplementary Information [file 41467_2019_8555_MOESM1_ESM.pdf]

Supplementary Information for

The flavonoid 4,4'-dimethoxychalcone promotes autophagy-dependent longevity across species

Carmona-Gutierrez et al.

Contents

Supplementary Figures 1-17
Supplementary Tables 1-6
Supplementary References

Supplementary Figure 1

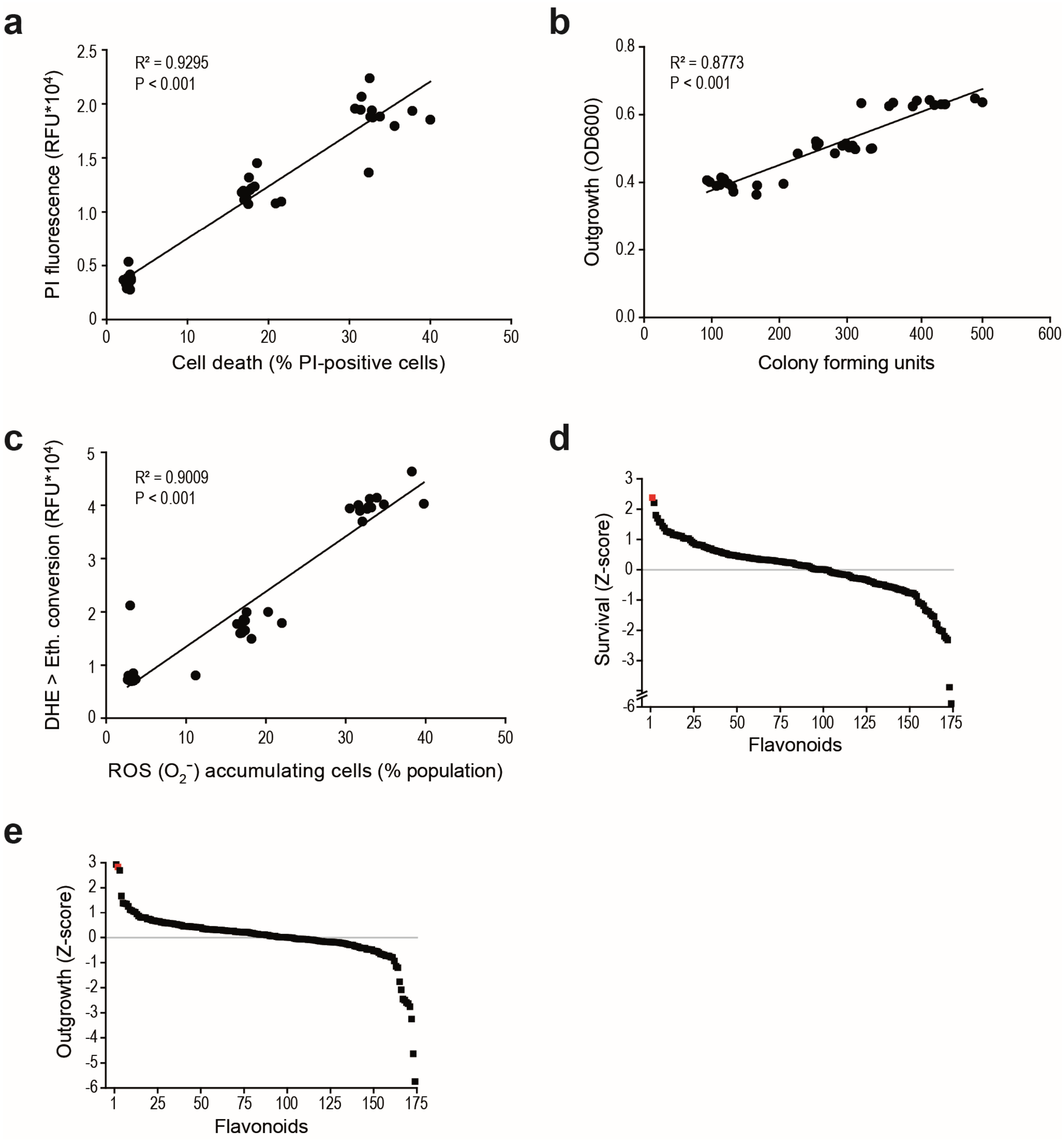

Supplementary Figure 1 (related to Figure 1). A high-throughput yeast screen for anti-ageing flavonoids unveils 4,4'-dimethoxychalcone as a cytoprotective substance.

(a-c) Correlation between the used high-throughput assays in the screen and established cell death and stress markers monitored in low scale experiments[1,2], i.e. propidium iodide (PI) staining (a), outgrowth capacity/clonogenicity (b) and DHE to ethidium conversion (b). Data was recorded using wild type yeast cells during chronological ageing (Linear regression coefficients are indicated. *P*-values indicate whether the slope significantly deviates from zero; ***$P < 0.001$). Source data are provided as a Source Data file.

(d-e) Z-scores of AUCs obtained for each flavonoid during the yeast chronological ageing screen for the PI staining (d) and outgrowth capacity (e) assays. Each data point represents one flavonoid. Note that this is the assay-specific data of these two fluorescent assays, which are plotted against each other in Fig. 1b. Source data are provided as a Source Data file.

Supplementary Figure 2

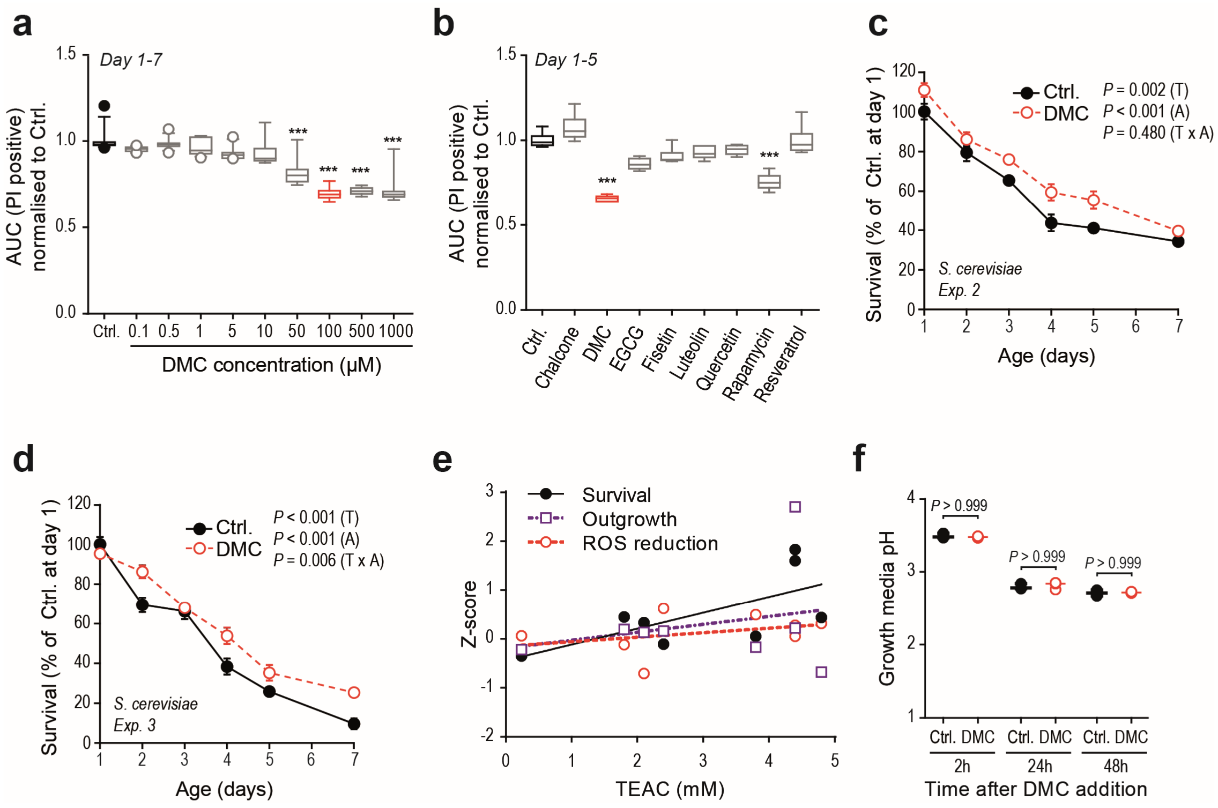

Supplementary Figure 2 (related to Figure 1). Yeast cells are protected during chronological ageing after a single treatment with 4,4'-dimethoxychalcone (DMC).

(a) Determination of the optimal cytoprotective dose of DMC in yeast. Wild type cells were treated with different concentrations of DMC or equivalent volume of DMSO (Ctrl.) and survival monitored throughout chronological ageing (days 1-7, $n = 12$ independent biological replicates) via PI staining and subsequent flow cytometry (30,000 cells were evaluated in each experiment). The resulting AUCs were normalised to the AUC of the control. Comparisons to Ctrl. by ANOVA/Bonferroni; ***P<0.001.

(b) Comparison of DMC with other commonly used cytoprotectors. Wild type cells were treated with 100 µM compound (except rapamycin: 40 nM) or equivalent volume of DMSO (Ctrl.) and survival monitored throughout chronological ageing (days 1-5) via PI staining and subsequent flow cytometry (30,000 cells evaluated in each experiment). $n = 6$ independent biological replicates. Comparisons to Ctrl. by ANOVA/Bonferroni; ***P<0.001. EGCG, Epigallocatechin gallate.

(c, d) Replicate ageing experiments showing survival determined by clonogenicity of yeast cells treated with 100 µM DMC or equivalent volume of DMSO (Ctrl.) during chronological ageing. Data represent means ± SEM ($n = 8$; P-values represent factor (T, treatment; A, age; T x A, interaction) comparisons by two-way ANOVA).

(e) Correlation between Survival, Outgrowth and ROS reduction Z-scores (according to Figure 1 b,c) with Trolox equivalent antioxidant activity (TEAC) based on data from Rice-Evans and Miller[3]. R^2 values of linear regression and P-values for testing whether slopes are significant non-zero are as follows: Survival, $R^2 = 0.4414$, $P = 0.0723$; Outgrowth, $R^2 = 0.0646$, $P = 0.5435$; ROS-reduction, $R^2 = 0.1259$, $P = 0.3884$.

(f) Yeast growth media pH after addition of DMC (100 µM) or equivalent amount of DMSO (Ctrl.). $n = 3$ independent biological replicates. Comparisons by ANOVA/Bonferroni.

Box plots represent IQR (line at median) and whiskers 10-90 percentile. Source data are provided as a Source Data file.

Supplementary Figure 3

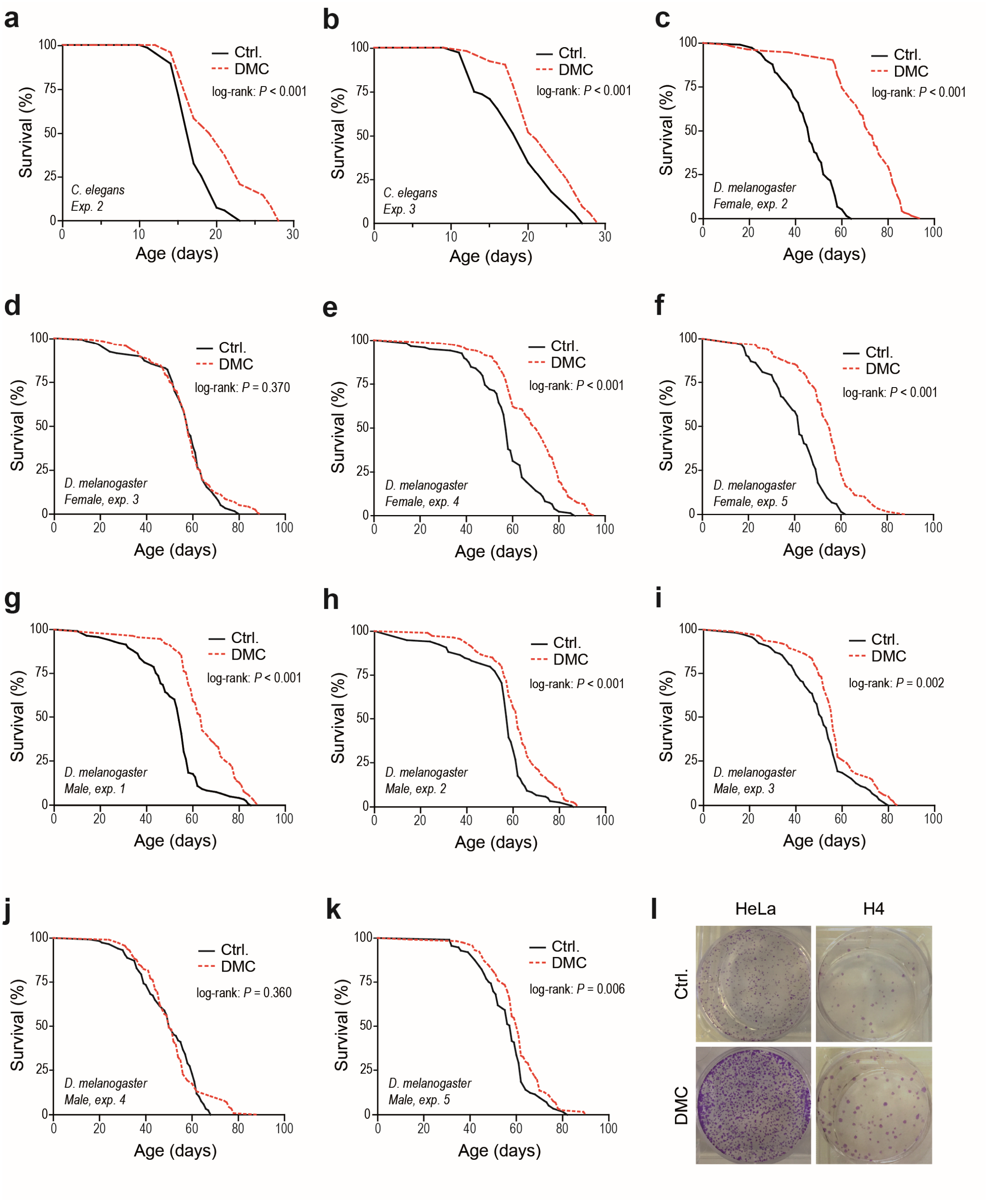

Supplementary Figure 3 (related to Figure 1). 4,4'-dimethoxychalcone (DMC) promotes survival in nematodes, flies, and human cells.

(a, b) Representative experiments showing survival of *C. elegans* during ageing with and without (Ctrl.) supplementation of food with DMC (41.6 µM) as young adults. For further details, refer to Supplementary Table 2.

(c-k) Replicate experiments showing survival of female (c-f) or male (g-k) *D. melanogaster* during ageing on Bloomington standard food with supplementation of DMC (200 µM) or equivalent amount of DMSO (Ctrl.). For further details, refer to Supplementary Table 2.

(l) Replicative viability of human HeLa and H4 cells upon treatment with 50 µM DMC as determined via yeast-like senescence assay. Representative images are shown.

Supplementary Figure 4

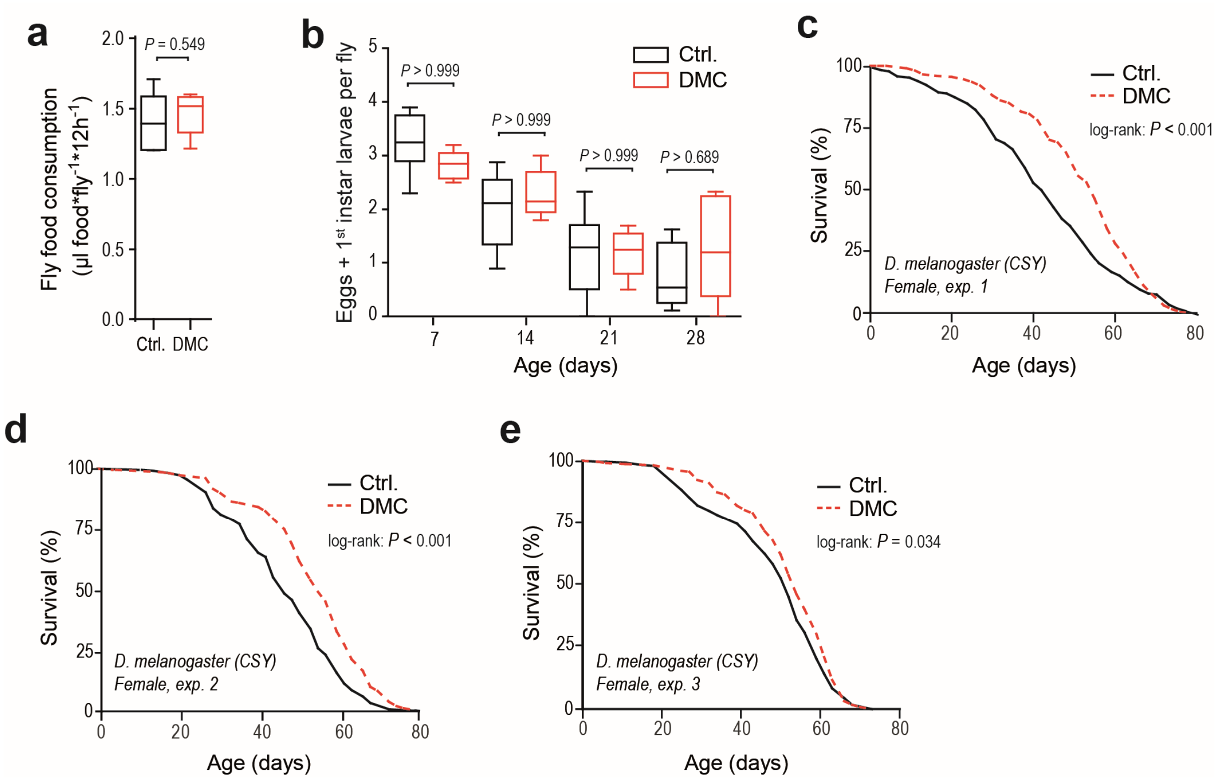

Supplementary Figure 4 (related to Figure 1). 4,4'-dimethoxychalcone (DMC) promotes survival in flies independently of food consumption and composition and without altering fly fecundity.

(a) Food consumption of wild type w^{1118} flies fed with liquid yeast extract-sucrose food with 200 µM DMC or equivalent amount of DMSO (Ctrl.) as measured by capillary feeding assay. $n = 5$ independent biological replicates; *P*-value represents comparison by two-sided *t*-test.

(b) Fecundity of w^{1118} flies fed with DMC (200 µM) or equivalent amount of DMSO (Ctrl.) over a period of three weeks. Data means ± SEM of eggs + 1st instar larvae laid per fly in a 16h interval. $n = 6$ independent biological replicates; *P*-values represent comparisons by repeated measures ANOVA/Bonferroni.

(c-e) Replicate experiments showing survival of female *D. melanogaster* during ageing on CSY food with supplementation of DMC (200 µM) or equivalent amount of DMSO (Ctrl.). For further details, refer to Supplementary Table 2.

Box plots represent IQR (line at median) and whiskers 10-90 percentile. Source data for (a-b) are provided as a Source Data file.

Supplementary Figure 5

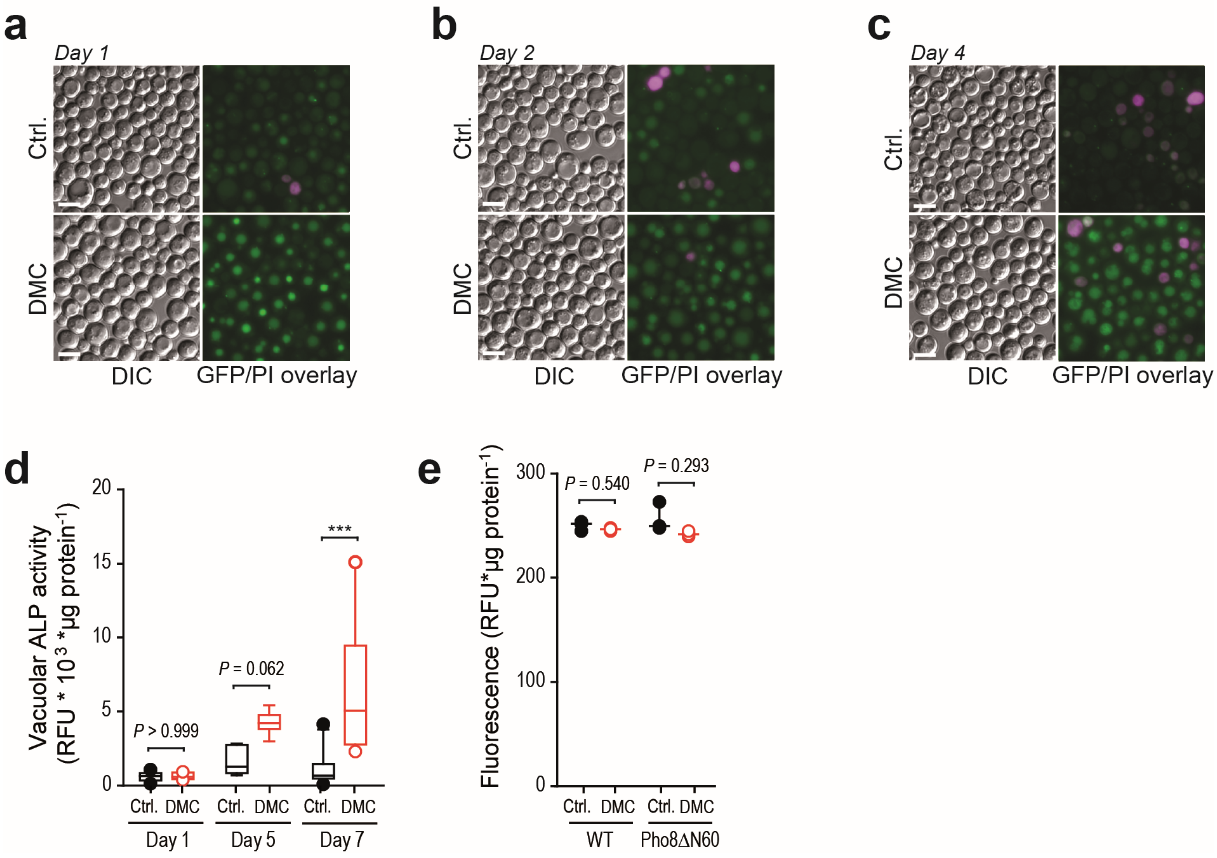

Supplementary Figure 5 (related to Figure 2). 4,4'-dimethoxychalcone (DMC) induces autophagy during yeast chronological ageing.

(a-c) Representative micrographs showing vacuolar accumulation of GFP-Atg8 (green) upon treatment with 100 µM DMC or equivalent amount of DMSO (Ctrl.) at days 1-4 of yeast chronological ageing; propidium iodide (PI) counterstaining served to visualize dead cells (magenta). Scale bars represent 5 µm.

(d) Yeast autophagic flux upon treatment with 100 µM DMC or equivalent amount of DMSO (Ctrl.) as determined via alkaline phosphatase (ALP) activity of Pho8ΔN60 strains during chronological ageing. $n = 11$ (day 1 and 7) or 7 (day 5) independent biological replicates, ***$P < 0.001$; P-values represent comparisons by repeated measures ANOVA/Bonferroni.

(e) ALP background fluorescence of protein extracts from yeast cells treated with 100 µM DMC or equivalent amount of DMSO (Ctrl.). $n = 3$ independent biological replicates; P-values represent comparisons by ANOVA/Bonferroni.

Box plots represent IQR (line at median) and whiskers 10-90 percentile. Source data for (d-e) are provided as a Source Data file.

Supplementary Figure 6

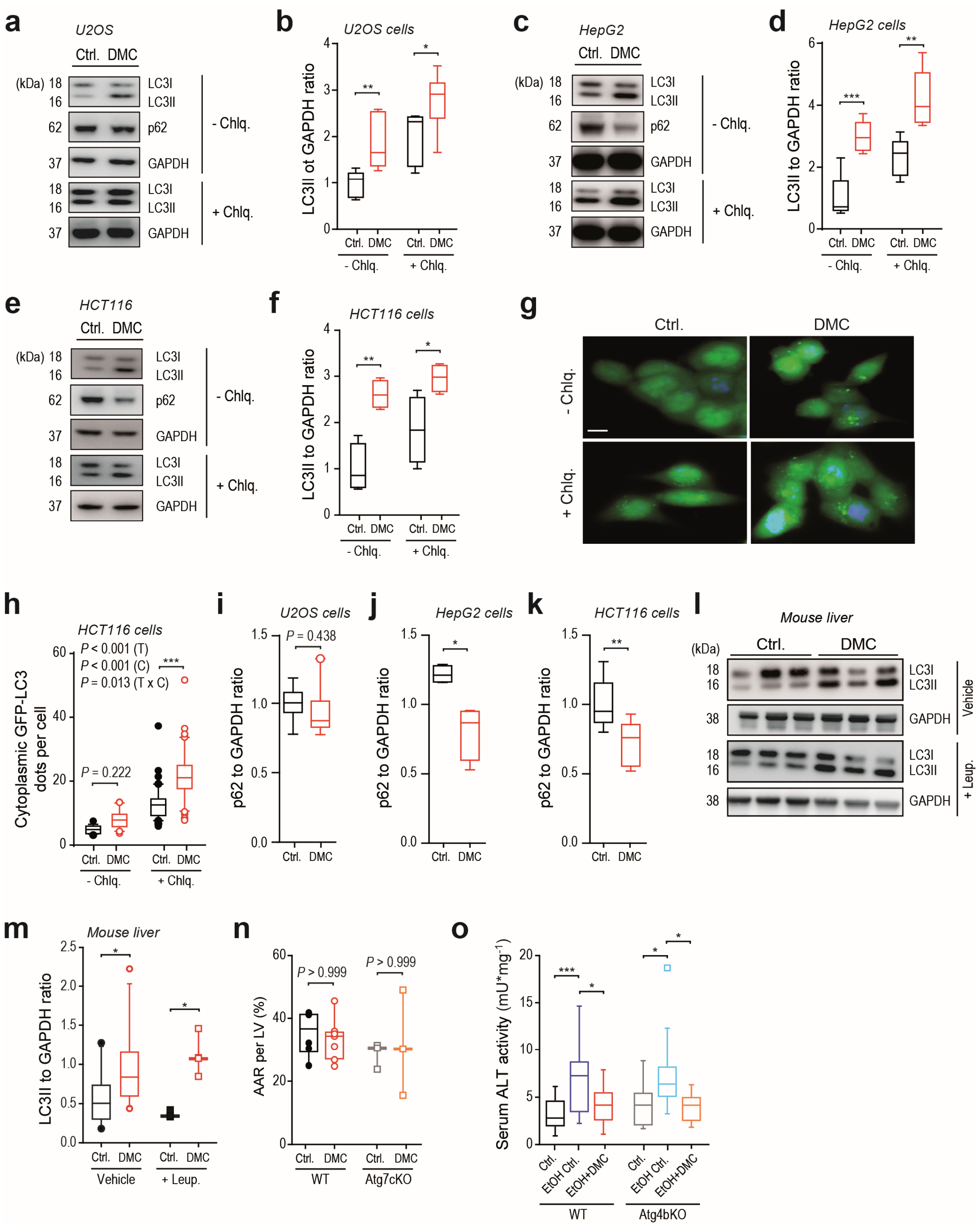

Supplementary Figure 6 (related to Figure 2 and Figure 3). 4,4'-dimethoxychalcone (DMC) induces autophagy in human cells and mice.

(a-k) Induction of autophagic flux in human U2OS, HepG2, and HCT116 cells 6h after treatment with 50 µM DMC or equivalent amount of DMSO (Ctrl.) as determined via western blot analysis of LC3 lipidation (LC3II) with or without chloroquine (a-f) and p62 levels without

chloroquine (i-k) or via videomicroscopy upon expression of GFP-LC3 with or without chloroquine (g, h). Representative blots for each cell line (a, c, e) and respective densitometric quantifications of LC3II to GAPDH (b, d, f) and p62 to GAPDH ratios (i-k) are shown. Representative pictures of videomicroscopic analysis of HCT116 cells (g) and quantification of GFP-LC3-positive dots (h) are depicted. Scale bar represents 10 µm. For b *n* = 6 (Ctrl.-Chlq.), 5 (Ctrl. +Chlq.), 9 (DMC -Chlq.), 7 (DMC +Chlq.); d *n* = 5; f *n* = 4; h *n* = 19 (Ctrl.-Chlq.), 20 (DMC -Chlq.), 50 (Ctrl. and DMC + Chlq.) ; i *n* = 6 (Ctrl.), 9 (DMC) ; j *n* = 5; k *n* = 4 independent biological replicates; *P*-values represent factor (T, treatment; C, chloroquine; T x C, interaction) by two-way ANOVA followed by simple main effects for (h) or comparisons to control by two-sided *t*-test for b, d, f and i-k).

(l-m) Autophagy induction in mouse liver tissue (m) determined by LC3 lipidation with leupeptin or vehicle injection after intraperitoneal injection of DMC (100 mg/kg) or DMSO (Ctrl.). *n* = 9 (Ctrl. Vehicle), 10 (DMC Vehicle), 3 (Leup.) animals, $^{*}P < 0.05$, $^{**}P < 0.01$. Comparisons by two-sided *t*-tests; (l) representative immunoblot.

(n) The size of the area at risk (AAR) per left ventricle after intraperitoneal injection of DMC (100 mg/kg) or equivalent amount of DMSO (Ctrl.) followed by prolonged ischemia. *n* = 6 (WT Ctrl.), 7 (WT DMC), 3 (Atg7cKO) animals; *P*-values represent comparison by ANOVA/Bonferroni.

(o) Serum alanine transaminase (ALT) activity following acute ethanol binge feeding after DMC or DMSO (Ctrl.) treatment as in (l-m) in wild type and whole-body Atg4 knockout mice. Comparisons by two-way ANOVA (T, treatment; EtOH, ethanol; T x EtOH, interaction) followed by Bonferroni-corrected simple main effects, $^{*}P < 0.05$, n = 17 (WT Ctrl. and EtOH Ctrl.), 16 (EtOH + DMC), 10 (ATG4BKO Ctrl.), 11 (ATG4BKO EtOH Ctrl. and EtOH+ DMC) animals.

Box plots represent IQR (line at median) and whiskers 10-90 percentile. Source data for (b, d, f, h-k, m-o) are provided as a Source Data file.

Supplementary Figure 7

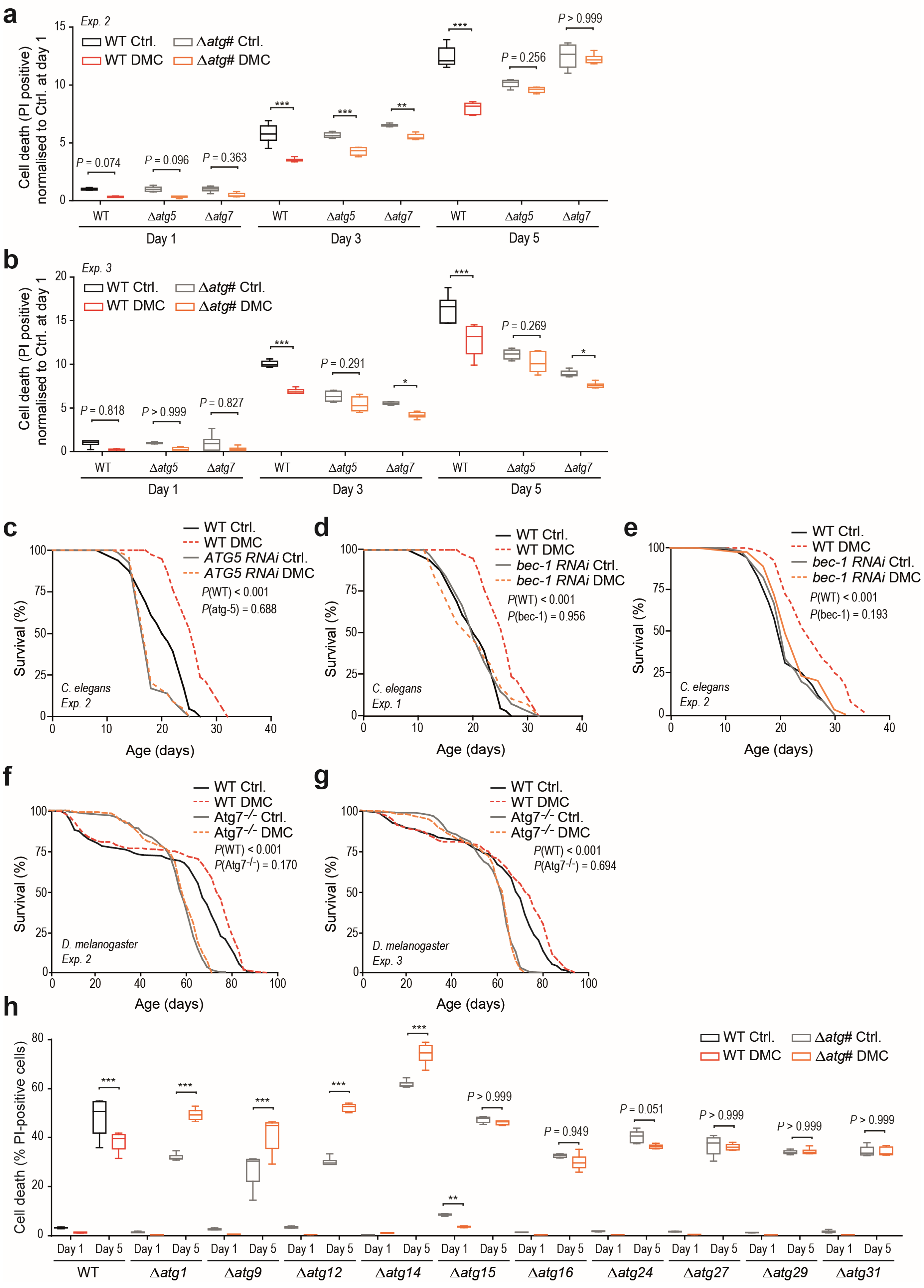

Supplementary Figure 7 (related to Figure 4). 4,4'-dimethoxychalcone (DMC)-mediated rescue during ageing depends on autophagy.

(a-b) Replicate experiments showing survival of *S. cerevisiae* wild type (control) and mutant strains deficient in either *ATG5* or *ATG7*, respectively, during chronological ageing and after treatment with 100 µM DMC or equivalent volume of DMSO (Ctrl.) as measured by flow cytometric analysis of PI staining. $n = 6$; $^{*}P < 0.05$, $^{**}P < 0.01$, $^{***}P < 0.001$; comparisons by two-way ANOVA for each day with strain and treatment as independent variables, followed by Bonferroni-corrected simple main effects.

(c-e) Representative experiments showing survival of wild type and autophagy-deficient *atg5* or *bec-1* RNAi *nematodes* during ageing with and without (Ctrl.) supplementation of food with DMC (41.6 µM). *P*-values represent Bonferroni-corrected pairwise comparisons log-rank analysis. For further details, refer to Supplementary Table 2.

(f-g) Replicate experiments showing survival of female wild type and *Atg7*-deficient mutant flies during ageing with supplementation of food with DMC (200 µM) or equivalent amount of DMSO (Ctrl.). *P*-values represent Bonferroni-corrected pairwise comparisons log-rank analysis. For further details, refer to Supplementary Table 2.

(h) Survival of *S. cerevisiae* wild type and mutant strains deficient in different ATGs at day 1 and day 5 of chronological ageing and after treatment with 100 µM DMC or equivalent volume of DMSO (Ctrl.) as measured by flow cytometric analysis of PI staining. $n = 7$ (WT), 5 (Δ*atg9*), 6 (all other mutants) independent biological replicates; $^{**}P = 0.005$, $^{***}P < 0.001$; comparisons by two-way ANOVA with strain and treatment as independent variables followed by Bonferroni-corrected simple main effects.

Box plots represent IQR (line at median) and whiskers 10-90 percentile. Source data for (a-b, h) are provided as a Source Data file.

Supplementary Figure 8

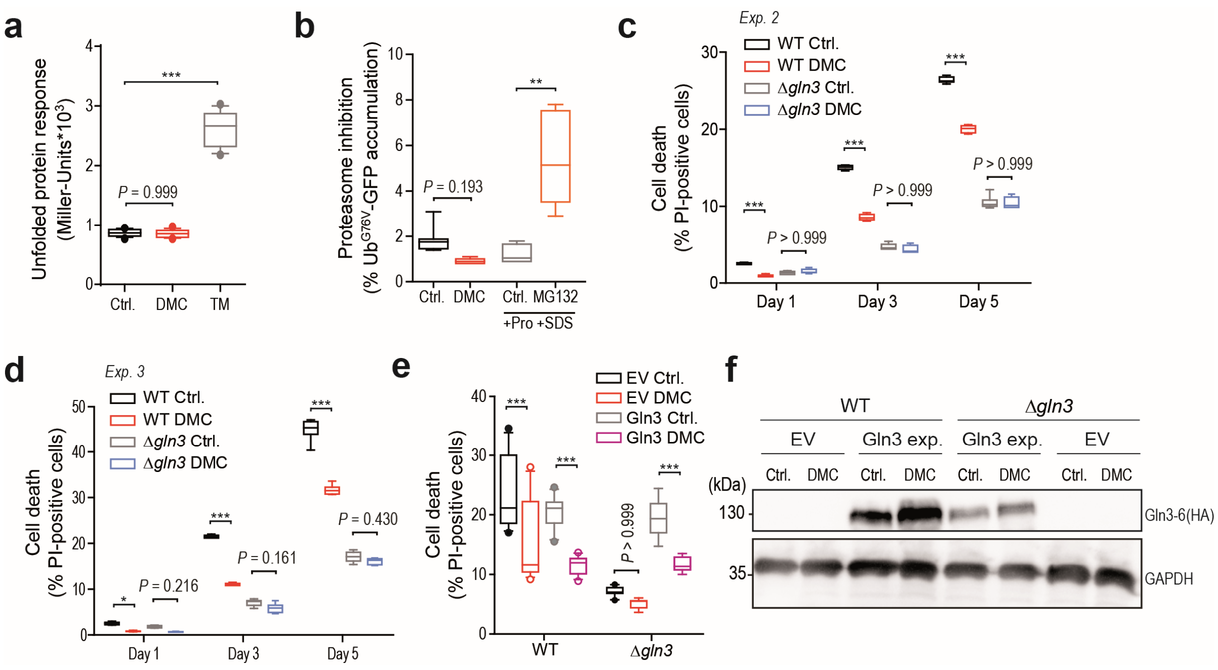

Supplementary Figure 8 (related to Figure 5). Gln3 is required for 4,4'-dimethoxychalcone (DMC)-mediated cytoprotection in yeast.

(a) Beta galacosidase activity of lacZ under the transcriptional control of unfolded protein response elements, indicative or ER-stress, 4h after treatment with 100 µM DMC, equivalent amount of DMSO (Ctrl.) or 5 µg/mL of the ER stressor tunicamycin (TM). *n* = 16 independent biological replicates; ****P* < 0.001, comparisons to Ctrl. by Kruskal-Wallis test/Dunn's multiple comparison test.

(b) Proteasome inhibition 4h after treatment with 100 µM DMC, equivalent amount of DMSO (Ctrl.) or 140 µM of the proteasome inhibitor MG132, as determined by flow cytometric measurement of the accumulation of proteasomal degradable UbG76V-GFP. Control media for MG132 was SC + 0.1% proline + 0.003% SDS to facilitate inhibitor uptake[4]. *n* = 8 independent biological replicates; ***P* < 0.01, compared to respective control by Kruskal-Wallis test/Dunn's multiple comparison test.

(c, d) Survival of *S. cerevisiae* wild type and *GLN3*-deficient mutant strains upon treatment with DMC (100 µM) or equivalent volume of DMSO (Ctrl.) during chronological ageing as measured via PI staining. Replicate experiments are shown. Note that Fig. 5B is based on data of (d). *n* = 6 independent biological replicates; **P* < 0.05, ****P* < 0.001; comparisons by two-way ANOVA for each day with strain and treatment as independent variables, followed by Bonferroni-corrected simple main effects.

(e) Survival of *S. cerevisiae* wild type and *GLN3*-deficient mutant strains either episomally expressing Gln3 (complementation) or carrying the corresponding empty vector control, respectively, as measured during chronological ageing via PI staining (day 3) upon treatment with 100 µM DMC or equivalent volume of DMSO (Ctrl.). *n* = 11 (Δ*gln3* EV DMC), 12 (all other conditions) independent biological replicates; ****P* < 0.001; comparisons by ANOVA/Bonferroni).

(f) Representative immunoblot of wild type and *GLN3*-deficient mutant yeast cells treated with DMC (100 µM) or DMSO (Ctrl.) and either episomally expressing HA-tagged Gln3 (complementation, Gln3 exp.) or carrying the corresponding empty vector control (EV), respectively. Expression was induced for 20h by addition of galactose (final concentration: 0.1%) and detection was performed using anti-HA and anti-GAPDH (loading control) antibodies.

Box plots represent IQR (line at median) and whiskers 10-90 percentile. Source data for (a-e) are provided as a Source Data file.

Supplementary Figure 9

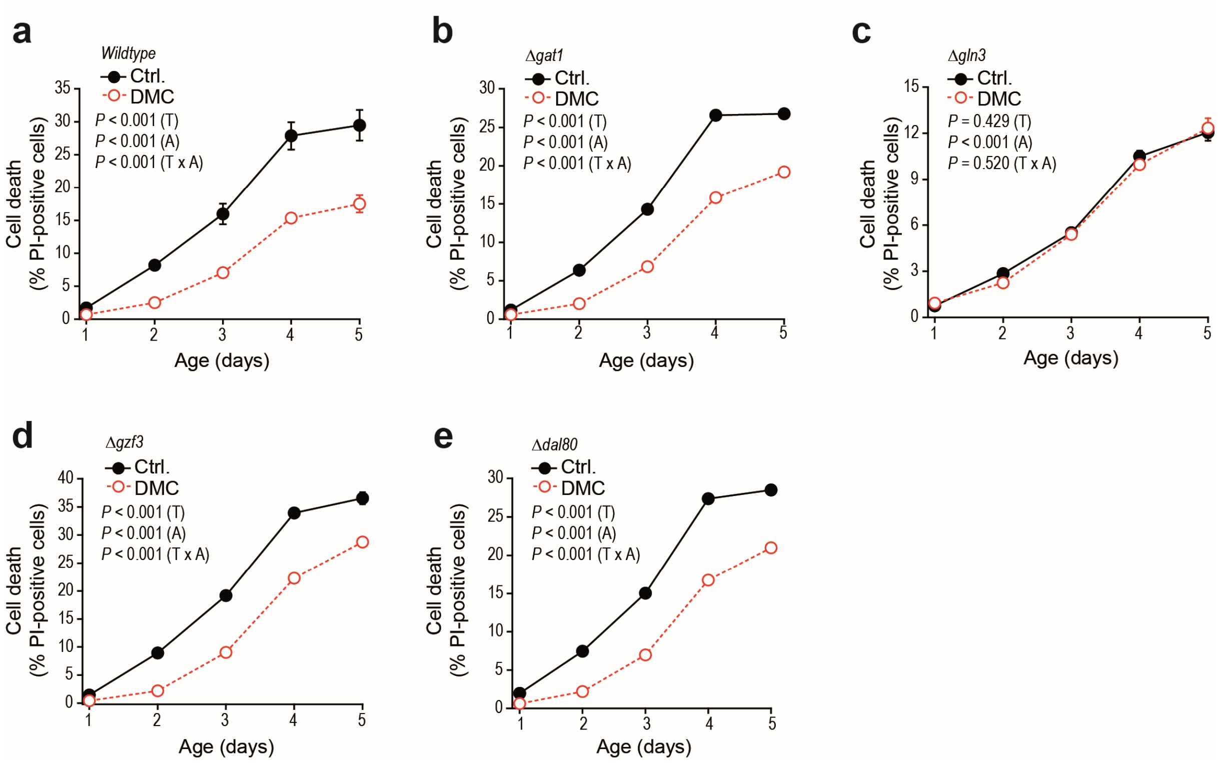

Supplementary Figure 9 (related to Figure 5). The effects of 4,4'-dimethoxychalcone (DMC) are dependent on Gln3, but not on the other yeast GATA transcription factors.

Ageing of wild type **(a)** or deletion mutants deficient in each of the yeast GATA factors, *GAT1* **(b)**, *GLN3* **(c)**, *GZF3* **(d)**, and *DAL80* **(e)**, treated with 100 µM DMC or equivalent amount of DMSO (Ctrl.). Data represent means ± SEM of a representative experiment (n = 5 (WT DMC at day 3), 6 (all other conditions) independent biological replicates; P-values represent factor (T, treatment; A, age; T x A, interaction) comparisons by two-way ANOVA). Source data are provided as a Source Data file.

Supplementary Figure 10

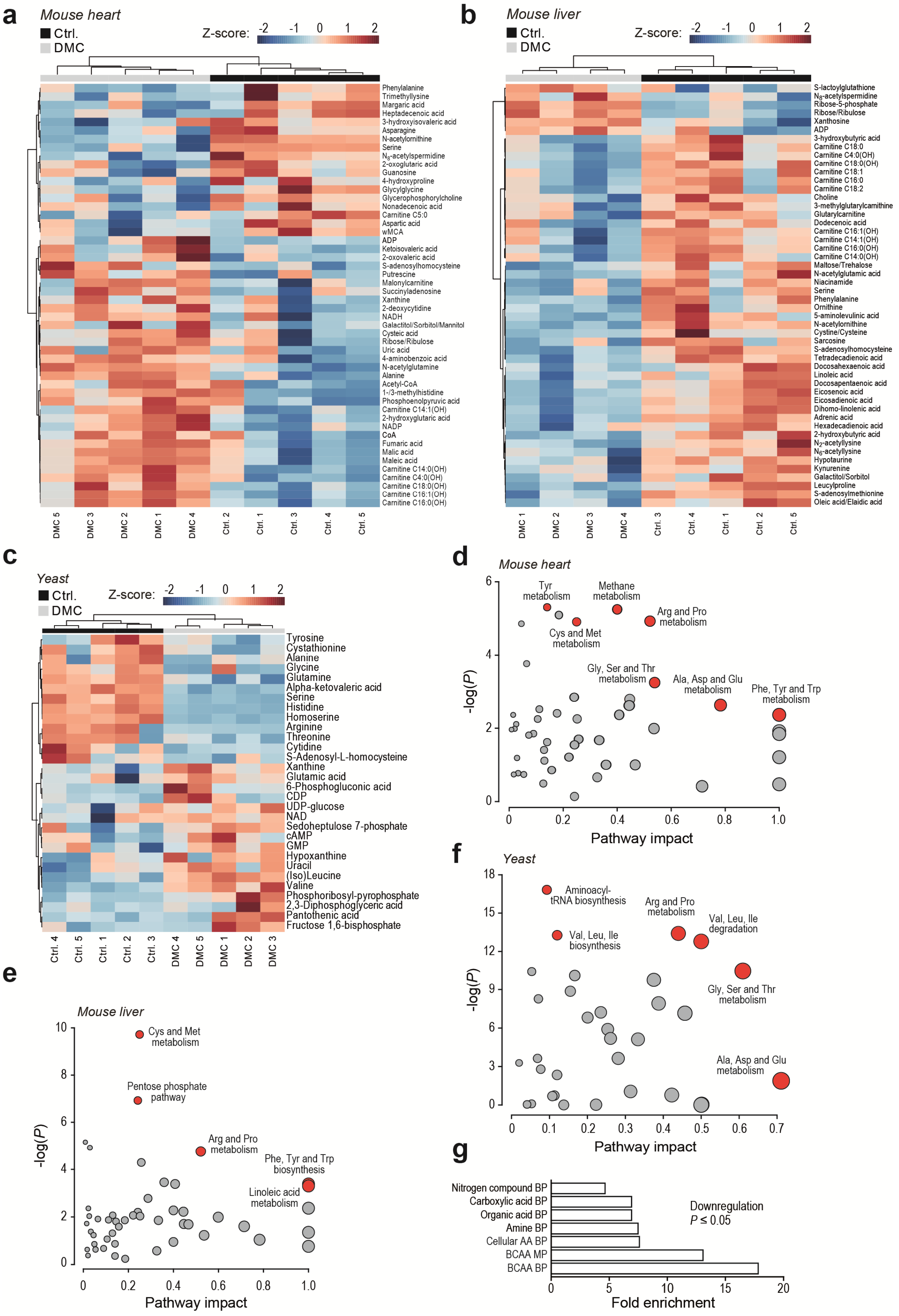

Supplementary Figure 10 (related to Figure 5). 4,4'-dimethoxychalcone (DMC) treatment impacts amino acid metabolism in mice.

(a-c) Pathway enrichment analysis of metabolites extracted from mouse hearts (a) or liver (b) 6h after intraperitoneal injection of DMC (100 mg/kg), or yeast 24h after DMC-treatment (100 µM). For (a-b) $n = 4$ (DMC liver), 5 animals; c, $n = 5$ independent biological replicates. Data represent the top 50 (a-b) or top 30 (c) metabolites ranked by *t*-tests, clustered hierarchical with Pearson distance measure and Ward clustering algorithm in MetaboAnalyst 3.0. Autoscaling was applied for features.

(d-f) Combined pathway topology (global test) and enrichment (relative-betweenness centrality) analysis of the data shown in (a-c) performed with MetaboAnalyst 3.0 using all compounds in the selected pathways as a reference.

(g) GO term enrichment analysis of significantly changed proteins of DMC-treated (100 µM) vs. DMSO-treated yeast cells. Note that enrichment was only significant for downregulated proteins. BP, biological process; MP, metabolic process; BCAA, branch-chained amino acids. Data represent means of 2 independent biological replicates.

Source data are provided as a Source Data file.

Supplementary Figure 11

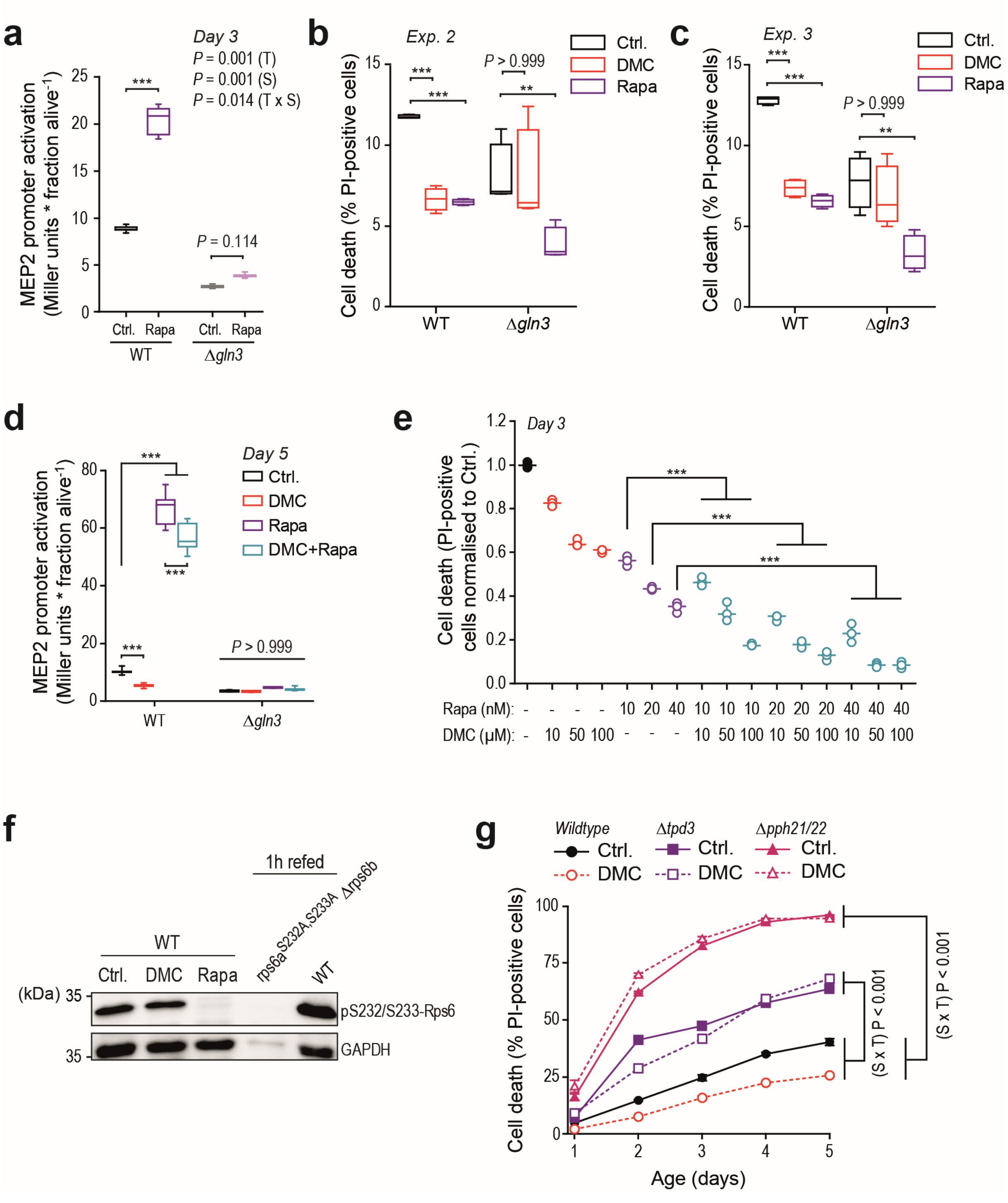

Supplementary Figure 11 (related to Figure 5 and Figure 6). Autophagy induction and cytoprotection by 4,4'-dimethoxychalcone and rapamycin follow distinct pathways.

(a) Gln3-dependent *MEP2* expression in yeast wild type and *GLN3*-deficient mutant strains upon treatment with 40 nM rapamycin (Rapa) or equivalent amount of DMSO (Ctrl.) as determined using a P_{MEP2}-*LacZ* fusion reporter at day 3 of chronological ageing. *n* = 6 independent biological replicates; *P*-values represent factor (T, treatment; S, strain; T x S, interaction) by two-way ANOVA followed by simple main effects ****P* < 0.001.

(b-c) Cell death of *S. cerevisiae* wild type and *GLN3*-deficient mutant strains upon treatment with DMC (100 µM), equivalent amount of DMSO (Ctrl.), or 40 nM rapamycin (Rapa) at day 3 of chronological ageing as measured via PI staining. Replicate experiments corresponding to Fig. 6a are shown. $n = 4$ independent biological replicates; comparisons by ANOVA/Bonferroni; $^{**}P < 0.01$, $^{***}P < 0.001$.

(d) MEP2 promoter activation using a P_{MEP2}-LacZ reporter in yeast wild type and *GLN3*-deficient mutant strains 5 days after treatment with DMC (50 µM), equivalent amount of DMSO (Ctrl.), 40 nM rapamycin (Rapa) or both compounds. $n = 6$ (Δ*gln3* Rapa), 12 (all other conditions) independent biological replicates. Comparisons by ANOVA/Bonferroni; $^{**}P < 0.01$, $^{***}P < 0.001$.

(e) Cell death of *S. cerevisiae* wild type and *GLN3*-deficient mutant strains at day 3 of chronological ageing upon treatment with DMC, rapamycin, or combinations thereof as measured via PI staining and flow cytometry. $n = 3$ independent biological replicates; comparisons by ANOVA/Tukey; $^{***}P < 0.001$.

(f) Representative immunoblot of Rps6 phosphorylation using a phospho-serine S232/233-specific antibody. Wild type cells were treated with DMC (100 µM), equivalent amount of DMSO (Ctrl.) or 40 nM rapamycin (Rapa) for 6h. As controls, stationary phase cultures of wild type or phosphorylation-deficient *rps6AS232/233* Δ*rps6B* mutant cells were refed for 1h with fresh SCD medium.

(g) Ageing of wild type or deletion mutants deficient in PP2A subunits *TPD3* and *PPH21/22* treated with 100 µM DMC or respective amount of DMSO (Ctrl.). Data represent means ± SEM of a representative experiment (n = 6 independent biological replicates; comparisons by three-way ANOVA using strain, time, and treatment as variables. *P*-values represent strain and treatment interaction).

Box plots represent IQR (line at median) and whiskers 10-90 percentile. Source data for (a-e, g) are provided as a Source Data file.

Supplementary Figure 12

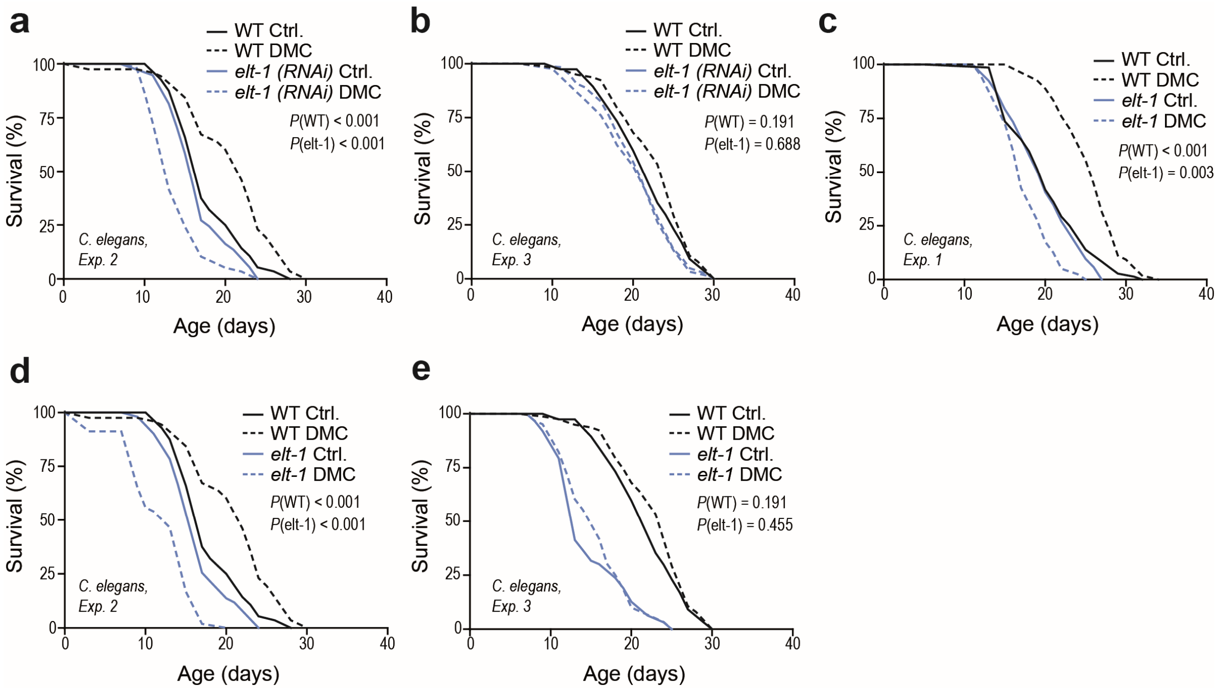

Supplementary Figure 12 (related to Figure 7). Deficiency of the *C. elegans* GATA factor *elt-1* abrogates the pro-autophagic and protective effects of 4,4'-dimethoxychalcone (DMC) in worms.

Survival of control and GATA-factor-deficient *elt-1* RNAi **(a, b)** or *elt-1* knockout **(c-e)** nematodes during ageing with and without (Ctrl.) supplementation of food with DMC (41.6 µM) compared to corresponding controls. Replicate ageing experiments with at least 80 worms per sample are shown. *P*-values represent Bonferroni-corrected pairwise comparisons by log-rank analysis. For further details, refer to Supplementary Table 2.

Supplementary Figure 13

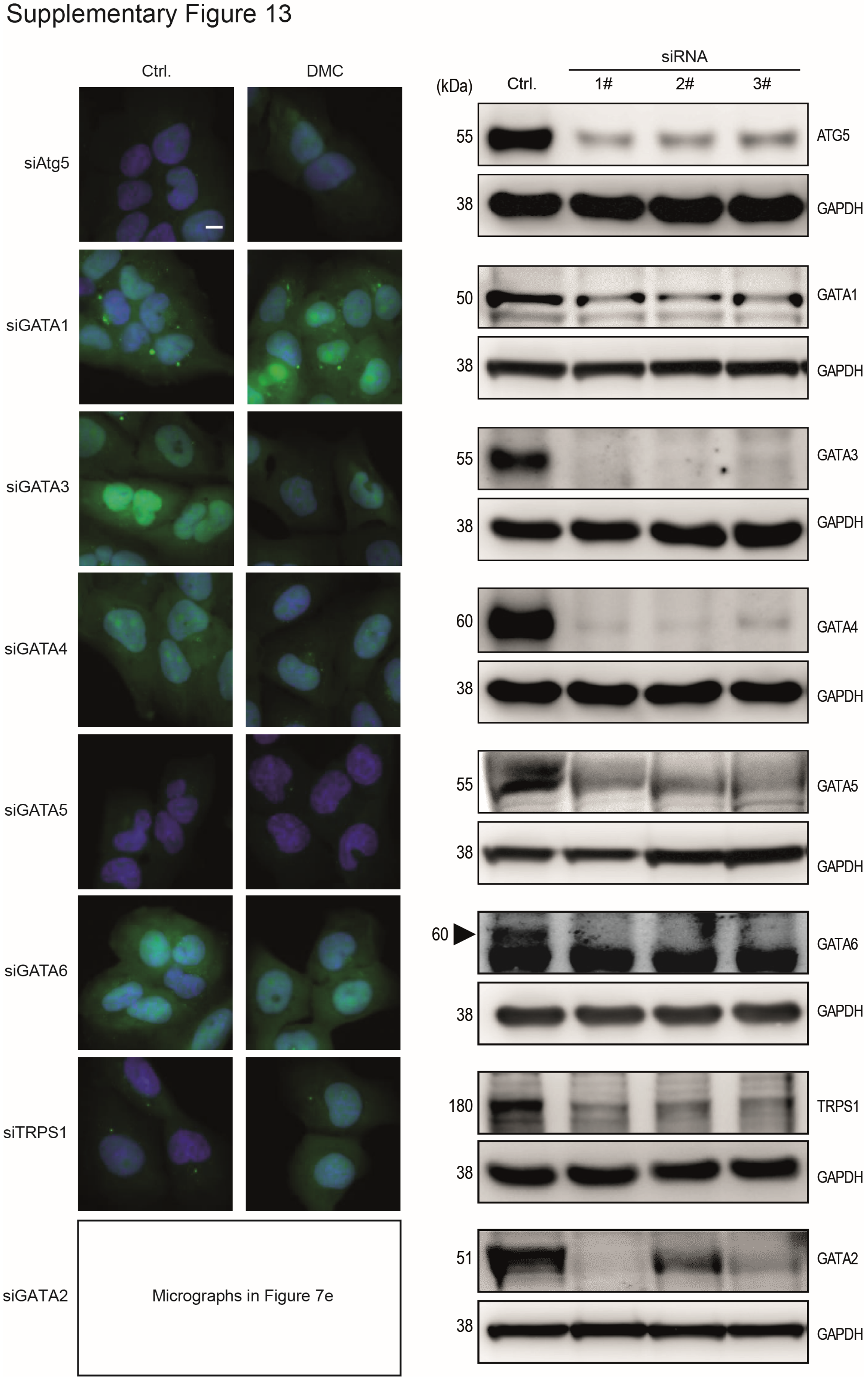

Supplementary Figure 13 (related to Figure 7). Silencing of specific GATA transcription factors in human cells abrogates the pro-autophagic potential of 4,4'-dimethoxychalcone.

Representative pictures and knockdown efficiency (as determined by western blot) corresponding to the videomicroscopic analysis shown in Fig. 7e of DMC-treated (50 µM) U2OS cells expressing GFP-LC3 and previously transfected with scramble siRNA (UNR) or siRNAs (sequence #2 for each target, according to Supplementary Table 5) targeting GATA1, GATA3, GATA4, GATA5, GATA6, TRPS1, and Atg5, respectively. Cell nuclei were stained with Hoechst 33342. Scale bar represents 10 µm.

Supplementary Figure 14

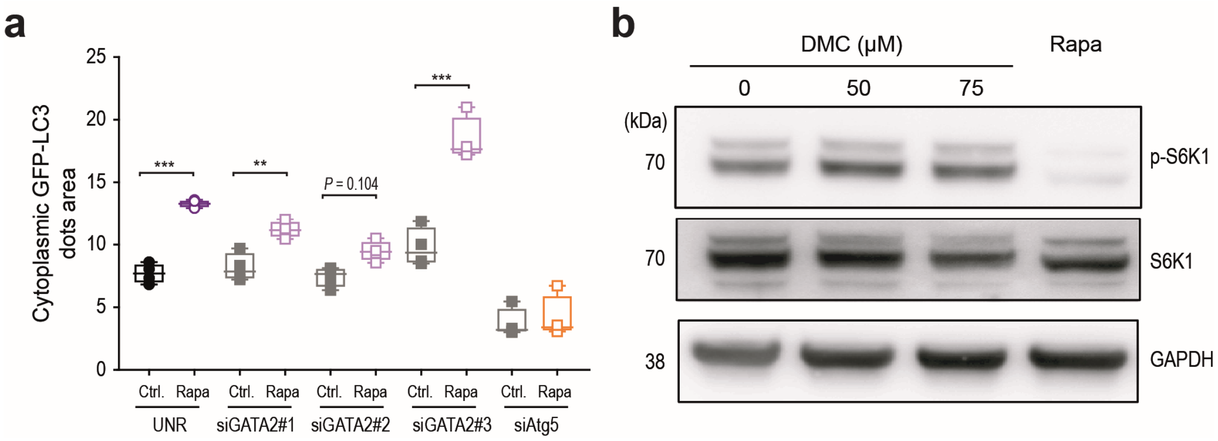

Supplementary Figure 14 (related to Figure 7). Differential effects of rapamycin and 4,4'-dimethoxychalcone on GATA-dependent autophagy induction and mTORC1 activity.

(a) Autophagy induction in rapamycin-treated (10 μM) human U2OS cells with siRNA against an unrelated sequence (UNR) or GATA2 or Atg5 as determined via videomicroscopy of cells expressing GFP-LC3. Cell nuclei were stained with Hoechst 33342. $^{**}P < 0.01$, $^{***}P < 0.001$, assessed by ANOVA/Bonferroni. $n = 4$ independent biological replicates.

(b) mTORC1-dependent S6K1 phosphorylation in U2OS cells 6h after treatment with different doses of DMC or 1 μM rapamycin, as assessed by immunoblotting using a threonine 389-specific phospho antibody.

Box plots represent IQR (line at median) and whiskers 10-90 percentile. Source data for (a) are provided as a Source Data file.

Supplementary Figure 15

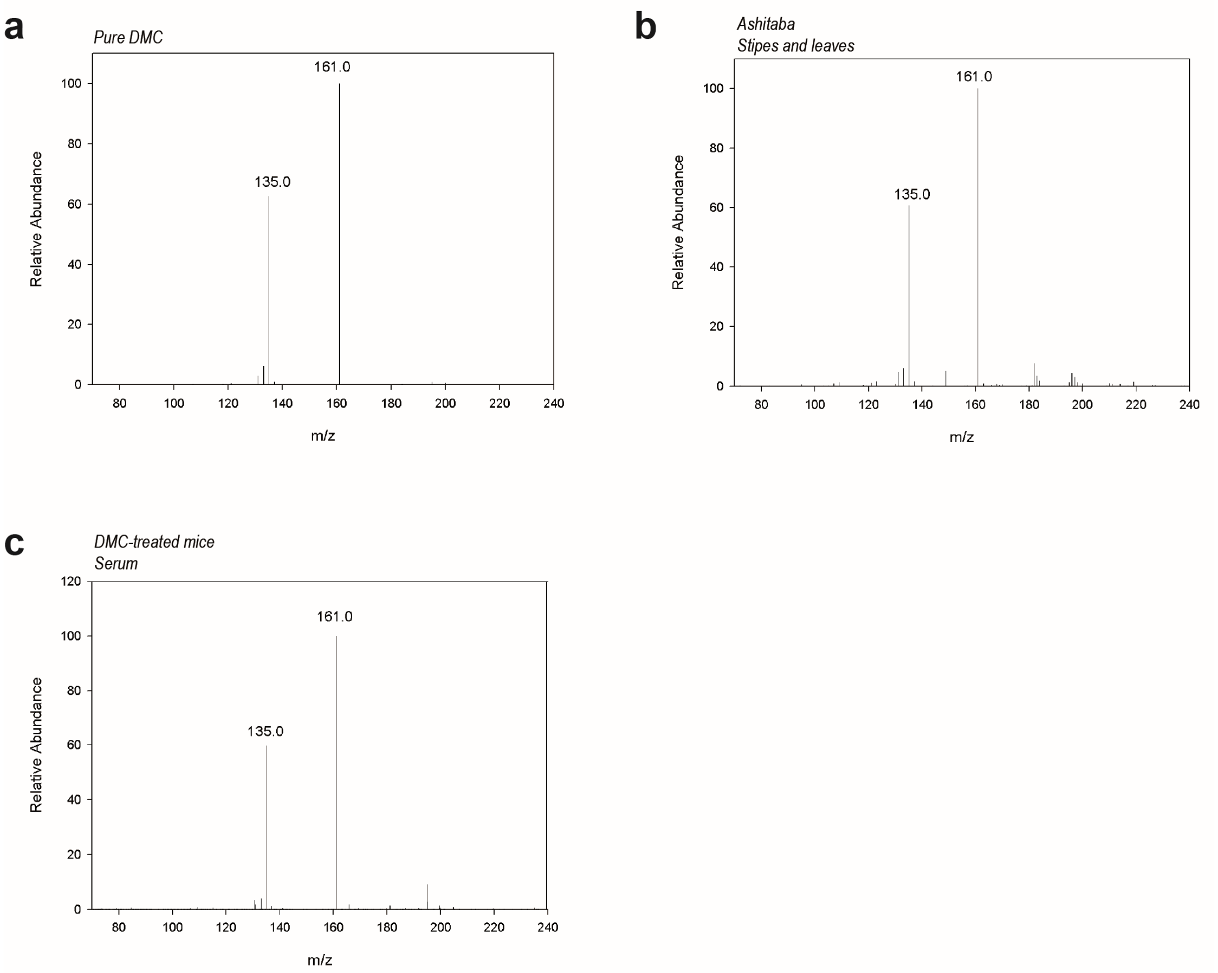

Supplementary Figure 15 (related to all Figures). 4,4'-dimethoxychalcone (DMC) is detectable in *Angelica keiskei koidzumi* (Ashitaba) as a natural source of the substance and in mice plasma upon animal feeding.

(a) Mass spectrometric signature of pure DMC (100 ng/mL) shown as its fragment ion spectrum, CID @35 (measured mass: 269.1169; ppm: -1.2).

(b) Representative fragment ion spectrum from HPLC-mass spectrometric analysis of plant extracts including stipes and leaves but without roots (measured mass: 269.1169; ppm: -1.2). Two independent extractions from two different plants were analysed. No DMC was detected in the root-extracts.

(c) Representative fragment ion spectrum from HPLC-mass spectrometric analysis of serum extracts from two 12-month old male mice fed with 0.25% DMC-supplemented food for 7 days (measured mass: 269.1179; ppm: 2.6). For comparison with fragment ion spectrum of standard DMC, refer to (a).

Supplementary Figure 16

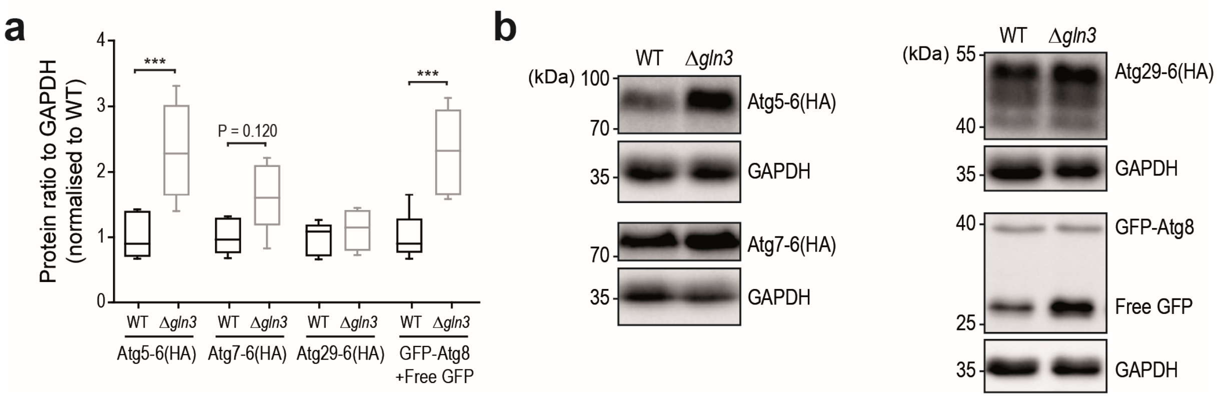

Supplementary Figure 16 (related to Figure 5). Atg protein levels are increased in *GLN3*-deficient yeast cells.

Quantification **(a)** and representative blots **(b)** of expression levels of autophagy related proteins (Atgs) 3 days after treatment with DMC (100 µM) or equivalent amount of DMSO (Ctrl.). Note that Atg5-6(HA) gets covalently linked to Atg12, resulting in an increased apparent molecular weight. $n = 5$ (GFP-Atg8), 6 (other Atgs); Comparisons by ANOVA/Bonferroni, $^{***}P < 0.001$.

Box plots represent IQR (line at median) and whiskers 10-90 percentile. Source data for (a) are provided as a Source Data file.

Supplementary Figure 17

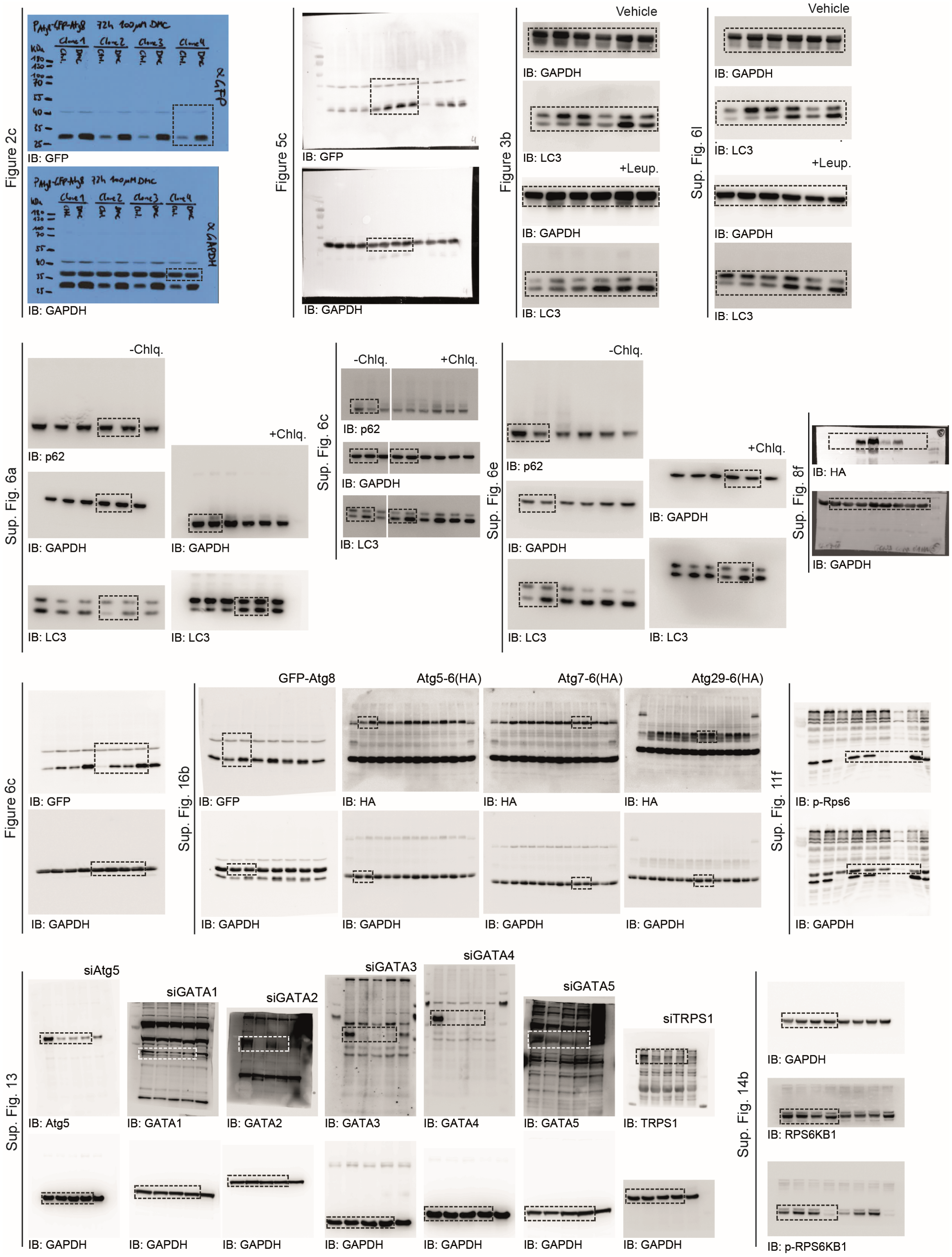

Supplementary Figure 17 (related to all Figures). Full scans of immunoblots.
Dotted lines indicate cropped blot areas.

Supplementary Tables

Supplementary Table 1 (related to Figure 1): List of tested flavonoids and screen results

Flavonoid	Subclass	Article no.
(-)-Epicatechin	Flavan-3-ol	0977 S
(-)-Epicatechin gallate	Anthocyan	0978S
(-)-Epigallocatechin	Flavan-3-ol	0979S
(-)-Epigallocatechingallate	Flavan-3-ol	0981 S
(-)-Homoeriodictyol	Flavanone	1283S
(+)-Catechin	Flavan-3-ol	0952
(+/-)-Equol	Isoflavonoid	1268
2,3-Dimethoxy-2'-hydroxychalcone	Chalcone	1263
2',4-Dihydroxy-4',6'-dimethoxychalcone	Chalcone	1282S
2',6'-Dihydroxy-4,4'-dimethoxychalcone	Chalcone	1042
2',6'-Dihydroxy-4-methoxychalcone-4'-O-neohesperidoside	Chalcone	1080
2'-Hydroxy-4,4',6'-trimethoxychalcone	Chalcone	1286
2'-Hydroxychalcone	Chalcone	1043
2'-Hydroxyflavanone	Flavanone	1271
2'-Methoxyflavone	Flavone	1270
3',4',5,5',6,7-Hexamethoxyflavone	Flavone	1180
3',4',5,7-Tetrahydroxy-3-methoxyflavone	Flavonol	1187
3',4',7,8-Tetrahydroxyflavone	Flavone	1269
3',4',7,8-Tetramethoxyflavone	Flavone	1342
3',4',7-Trihydroxyflavone	Flavone	1307
3',4',7-Trimethoxyflavone	Flavone	1308
3',4'-Dihydroxyflavone	Flavone	1309
3',4'-Dimethoxyflavone	Flavone	1204
3,5,7-Trihydroxy-3',4',5'-trimethoxyflavone	Flavonol	1294
3',5,7-Trihydroxy-3,4'-dimethoxyflavone	Flavonol	1297
3,6-Dihydroxyflavone	Flavone	1310
3,7-Dihydroxy-3',4',5'-trimethoxyflavone	Flavone	1023
3-Methoxyflavone	Flavone	1345
4,4'-Dimethoxychalcone	Chalcone	1293
4',6,7-Trihydroxyisoflavone	Isoflavonoid	1188
4',6,7-Trimethoxyisoflavone	Isoflavonoid	1295
4',7-Dimethoxyisoflavone	Isoflavonoid	1052
4-Deoxyphloridzin	Chalcone	1291
4'-Hydroxychalcone	Chalcone	1299
4-Hydroxychalcone	Chalcone	1252
4'-Hydroxyflavanone	Flavanone	1181
4'-Methoxychalcone	Chalcone	1217
4-Methoxychalcone	Chalcone	1216
4'-Methoxyflavanone	Flavanone	1185
5,6-Benzoflavone	Flavone	1007
5,7-Dimethoxyflavonone	Flavanone	1296
5-Methoxyflavanone	Flavanone	1186
5-Methoxyflavone	Flavone	1020
5-Methyl-7-methoxyisoflavone	Isoflavonoid	1329
6,7 Dihydroxyflavone	Flavone	1012
6-Hydroxyflavanone	Flavanone	1182
6-Methoxyflavanone	Flavanone	1079
6-Methoxyflavone	Flavone	1062
6-Methoxyflavonol	Flavonol	1193
6-Methoxyluteolin	Flavone	1084
7,8-Benzoflavone	Flavone	1008

7,8-Dimethoxyflavone	Flavone	1175
7-Hydroxy-5-methylflavone	Flavone	1149
7-Hydroxyflavanone	Flavanone	1212
7-Hydroxyflavonol	Flavonol	1258
7-Methoxyflavone	Flavone	1289
7-Methoxyflavonol	Flavonol	1194
Apigenin-4',5,7-trimethylether	Flavone	1324
Apigenin-7-O-glucoside	Flavone	1004S
Apigeninidin chloride	Anthocyan	0921S
Baicalein-5,6,7-trimethylether	Flavone	1323
Baicalein-7-methylether	Flavone	1250
Baicalin	Flavone	1280S
Bavachinin	Flavanone	1237S
Butein	Chalcone	1103S
Callistephin chloride	Anthocyan	0907S
Chalcone	Chalcone	1041
Chrysoeriol	Flavone	1104S
Cupressuflavone	Flavone	1281S
Cyanidin chloride	Anthocyan	0909S
Cyanidin-3-O-lathyroside chloride	Anthocyan	0936S
Cyanidin-3-O-sophoroside chloride	Anthocyan	0937S
Cyanin chloride	Anthocyan	0932S
Datiscetin	Flavonol	1141S
Datiscin	Flavonol	0086
Delphin chloride	Anthocyan	0941S
Delphinidin chloride	Anthocyan	0904S
Didymin	Flavanone	1166S
Dihydromyricetin	Flavonol	1351
Dihydrorobinetin	Flavonol	1037
Diosmetin	Flavone	1108S
Eriocitrin	Flavanone	1110S
Eriodictyol	Flavanone	0056
Eupatorin	Flavone	1014
Eupatorin-5-methylether	Flavone	1015
Fisetin	Flavonol	0068
Fisetinidin chloride	Anthocyan	0926S
Flavanomarein	Flavanone	0058S
Flavanone	Flavanone	1038
Flavone	Flavone	1016
Fustin	Flavonol	1146
Gardenin A	Flavone	1339S
Genistein-4',7-dimethylether	Isoflavonoid	1177
Genistein-7-O-glucoronide	Isoflavonoid	1298
Genistin	Isoflavonoid	1350
Geraldol	Flavonol	1325S
Glycitein	Isoflavonoid	1168S
Glycitin	Isoflavonoid	1317
Hamamelitannin	Flavan-3-ol	1318
Hinokiflavone	Flavone	1017
Homobutein	Chalcone	1117S
Homoeriodictyol	Flavanone	0091
Ideain chloride	Anthocyan	0923S
Ipriflavone	Isoflavonoid	1328
Isoliquiritigenin	Chalcone	1279
Isorhamnetin	Flavonol	0065
Isorhamnetin-3-O-glucoside	Flavonol	1228
Isorhamnetin-3-O-rutinoside	Flavonol	1333S
Isorhoifolin	Flavone	1121S

Isosakuranetin	Flavanone	0090
Isovitexin	Flavone	1235S
Kaempferide	Flavonol	0063
Kaempferol-3,4',7-trimethylether	Flavonol	1070
Kaempferol-3-O-glucoside	Flavonol	1243S
Kaempferol-3-O-glucuronide	Flavonol	1316
Kaempferol-3-O-rutinoside	Flavonol	1053S
Kaempferol-7-O-neohesperidoside	Flavonol	1069
Karanjin	Flavonol	1071
Keracyanin chloride	Anthocyan	0914S
Kuromanin chloride	Anthocyan	0915S
Laricitrin	Flavonol	1340S
Liquiritigenin	Flavanone	1150S
Luteolin	Flavone	0052
Luteolin tetramethylether	Flavone	1275
Luteolin-3',7-di-O-glucoside	Flavone	0085S
Luteolin-4'-O-glucoside	Flavone	1083
Luteolin-7-O-glucoside	Flavone	0053
Luteolinidin chloride	Anthocyan	0925S
Malvidin chloride	Anthocyan	0913S
Malvin chloride	Anthocyan	0930S
Marein	Chalcone	0059S
Maritimein	Auron	0060S
Myricitrin	Flavonol	1029S
Myrtillin chloride	Anthocyan	0938
Naringenin-7-O-glucoside	Flavanone	1090
Naringin	Flavanone	1129S
Narirutin	Flavanone	1130S
Neodiosmin	Flavone	1249
Neoeriocitrin	Flavanone	1131S
Neohesperidin dihydrochalcone	Chalcone	1231
Oenin chloride	Anthocyan	0911S
Ononin	Isoflavonoid	1241S
Orientin	Flavone	1054S
Pelargonidin chloride	Anthocyan	0912S
Pelargonin chloride	Anthocyan	0903
Peonidin chloride	Anthocyan	0906S
Phloretin	Chalcone	1044
Phloridzin	Chalcone	1046
Pinocembrin	Flavanone	1162
Pinocembrin-7-methylether	Flavanone	1095
Poncirin	Flavanone	1133S
Pratol	Flavone	1134S
Prunetin	Isoflavonoid	1051
Quercetagetin	Flavonol	1030
Quercetin-3,4,7,3',4'-pentamethylether	Flavonol	1341S
Quercetin-3,4'-di-O-glucoside	Flavonol	1285
Quercetin-3-O-glucopyranoside	Flavonol	1347S
Quercetin-3-O-glucose-6''-acetate	Flavonol	1327S
Quercitrin	Isoflavonoid	1099
Resveratrol	Stilbenoid	1236S
Rhamnazin	Flavonol	1305S
Rhamnetin	Flavonol	1136S
Robinetinidin chloride	Anthocyan	0927S
Robinin	Flavonol	1032
Saponarin	Flavone	1238S
Sieboldin	Chalcone	1358S
Sissotrin	Isoflavonoid	1244S

Spiraeoside	Flavonol	1201
Syringetin	Flavonol	1239S
Tricetin	Flavone	1335S
Trilobatin	Chalcone	1314S
Xanthohumol	Chalcone	1346S

The names of flavonoids used for the initial screen in this study are alphabetically listed together with their corresponding subclasses according to Croizier et al.[5]. All substances were purchased from Extrasynthese (France); see depicted article no. Marked in green is the top hit that was further analysed in the present study. *AUC of day 1-5 normalised to DMSO control. **Endpoint OD_{600} at day 3 normalised to DMSO control.

Supplementary Table 2 (related to all Figures): Worm and fly lifespan analysis overview

Strain	Figure	No. of animals	Censored	Median lifespan	Hazard ratio	95% CI
C. elegans						
WT + DMSO	Fig. 1g	71	6	19	2.422	1.603 to 3.658
WT + DMC	Fig. 1g	70	18	23		
WT + DMSO	Sup. Fig. 3a	80	13	17	2.813	1.802 to 4.391
WT + DMC	Sup. Fig. 3a	81	33	20		
WT + DMSO	Sup. Fig. 3b	80	8	19	1.983	1.335 to 2.947
WT + DMC	Sup. Fig. 3b	80	28	21		
WT + DMSO	Fig. 4b	60	8	20	2.981	1.853 to 4.796
WT + DMC	Fig. 4b	60	20	25		
atg-5 (RNAi) + DMSO	Fig. 4b	50	13	20	1.114	0.6572 to 1.889
atg-5 (RNAi) + DMC	Fig. 4b	50	15	20		
WT + DMSO	Sup. Fig. 7i	same as Fig. 4b				
WT + DMC	Sup. Fig. 7i	same as Fig. 4b				
bec-1 (RNAi) + DMSO	Sup. Fig. 7i	60	9	21	1.369	0.8528 to 2.199
bec-1 (RNAi) + DMC	Sup. Fig. 7i	48	13	21		
WT + DMSO	Sup. Fig. 7g	80	16	21	4.055	2.552 to 6.442
WT + DMC	Sup. Fig. 7g	63	25	26		
atg-5 (RNAi) + DMSO	Sup. Fig. 7g	80	15	17	1.098	0.6948 to 1.736
atg-5 (RNAi) + DMC	Sup. Fig. 7g	56	12	17		
WT + DMSO	Sup. Fig. 7h	same as Sup. Fig. 7g				
WT + DMC	Sup. Fig. 7h	same as Sup. Fig. 7g				
bec-1 (RNAi) + DMSO	Sup. Fig. 7h	80	12	20	1.013	0.6350 to 1.617
bec-1 (RNAi) + DMC	Sup. Fig. 7h	56	26	19		
WT + DMSO	Fig. 7a	80	8	20	2.272	2.030 to 4.184
WT + DMC	Fig. 7a	75	21	26		
elt-1 (RNAi) + DMSO	Fig. 7a	80	14	21	1.048	0.721 to 1.558
elt-1 (RNAi) + DMC	Fig. 7a	80	38	21		
WT + DMSO	Sup. Fig. 13a	80	24	17	1.876	1.566 to 3.400
WT + DMC	Sup. Fig. 13a	80	22	22		
elt-1 (RNAi) + DMSO	Sup. Fig. 13a	80	24	16	0.5272	0.2776 to 0.5877
elt-1 (RNAi) + DMC	Sup. Fig. 13a	80	22	13		
WT + DMSO	Sup. Fig. 13b	80	8	22	1.213	0.9195 to 1.760
WT + DMC	Sup. Fig. 13b	85	9	24		
elt-1 (RNAi) + DMSO	Sup. Fig. 13b	85	4	21	1.003	0.7197 to 1.399
elt-1 (RNAi) + DMC	Sup. Fig. 13b	85	24	21		
WT + DMSO	Sup. Fig. 13c	same as Fig. S13a				
WT + DMC	Sup. Fig. 13c	same as Fig. S13a				
elt-1 + DMSO	Sup. Fig. 13c	80	29	16	0.4356	0.1955 to 0.4395
elt-1 + DMC	Sup. Fig. 13c	80	24	13		
WT + DMSO	Sup. Fig. 13d	same as Fig. 3e				
WT + DMC	Sup. Fig. 13d	same as Fig. 3e				
elt-1 + DMSO	Sup. Fig. 13d	80	28	20	0.5759	0.3036 to 0.7365
elt-1 + DMC	Sup. Fig. 13d	77	37	17		
WT + DMSO	Sup. Fig. 13e	same as Fig. S13b				
WT + DMC	Sup. Fig. 13e	same as Fig. S13b				
elt-1 + DMSO	Sup. Fig. 13e	70	7	13	1.129	0.6043 to 1.226
elt-1 + DMC	Sup. Fig. 13e	76	16	15		
D. melanogaster						
WT + DMSO (♀)	Fig. 1h	115	5	58	3.515	2.567 to 4.812
WT + DMC (♀)	Fig. 1h	116	4	68		
WT + DMSO (♀)	Fig. 4c	pool of WT from Fig. 1h and Sup. Fig. 3c-f				
WT + DMC (♀)	Fig. 4c	pool of WT from Fig. 1h and Sup. Fig. 3c-f				
Atg7-/- + DMSO (♀)	Fig. 4c	90	0	49	0.7802	0.5529 to 0.9886
Atg7-/- + DMC (♀)	Fig. 4c	94	0	48		
WT + DMSO (♀)	Sup. Fig. 3c	115	5	46	13.14	9.025 to 19.14
WT + DMC (♀)	Sup. Fig. 3c	114	6	72		

WT + DMSO (♀)	Sup. Fig. 3d	117	3	58	1.134	0.8612 to 1.494
WT + DMC (♀)	Sup. Fig. 3d	116	4	58		
WT + DMSO (♀)	Sup. Fig. 3e	118	2	57	2.714	2.016 to 3.653
WT + DMC (♀)	Sup. Fig. 3e	117	3	70		
WT + DMSO (♀)	Sup. Fig. 3f	116	4	42	3.571	2.611 to 4.884
WT + DMC (♀)	Sup. Fig. 3f	117	3	55		
WT + DMSO (♂)	Sup. Fig. 3g	118	2	54	2.237	2.256 to 3.939
WT + DMC (♂)	Sup. Fig. 3g	115	5	64		
WT + DMSO (♂)	Sup. Fig. 3h	119	1	58	1.863	1.402 to 2.475
WT + DMC (♂)	Sup. Fig. 3h	114	6	62		
WT + DMSO (♂)	Sup. Fig. 3i	118	2	51.5	1.565	1.136 to 2.064
WT + DMC (♂)	Sup. Fig. 3i	116	4	56		
WT + DMSO (♂)	Sup. Fig. 3j	117	3	50	1.141	0.8620 to 1.510
WT + DMC (♂)	Sup. Fig. 3j	114	6	50		
WT + DMSO (♂)	Sup. Fig. 3k	116	4	58	1.487	1.122 to 1.972
WT + DMC (♂)	Sup. Fig. 3k	117	3	60		
WT + DMSO (♀)	Sup. Fig. 7j	235	9	67	1.422	1.282 to 1.868
WT + DMC (♀)	Sup. Fig. 7j	228	10	75		
Atg7-/- + DMSO (♀)	Sup. Fig. 7j	232	9	59	1.122	0.9617 to 1.395
Atg7-/- + DMC (♀)	Sup. Fig. 7j	233	11	59		
WT + DMSO (♀)	Sup. Fig. 7k	226	10	70	1.352	1.192 to 1.738
WT + DMC (♀)	Sup. Fig. 7k	239	8	74		
Atg7-/- + DMSO (♀)	Sup. Fig. 7k	233	6	62	0.9677	0.7927 to 1.156
Atg7-/- + DMC (♀)	Sup. Fig. 7k	224	17	64		

The results of all ageing experiments performed in *C. elegans* and *D. melanogaster* are depicted (including the corresponding figure numbers), detailing the total and censored number of animals as well as the obtained median lifespans. In addition, statistical specifications for all experiments are provided, including the hazard ratio and the 95% confidence interval (CI) upon comparison of DMSO (Ctrl.) and DMC treatment.

Supplementary Table 3 (related to all Figures): *S. cerevisiae* yeast strains used in this study

Yeast strain	Genotype	Origin/Reference
BY4742 (WT)	MATα *his3Δ-1 leu2Δ-0 lys2Δ-0 ura3Δ-0*	Euroscarf
Δ*atg1*	BY4742 *atg1Δ::URA3*	This study [a]
Δ*atg5*	BY4742 *atg5Δ::URA3*	This study [a]
Δ*atg7*	BY4742 *atg7Δ::URA3*	This study [a]
Δ*atg9*	BY4742 *atg9Δ::kanMX*	Euroscarf
Δ*atg12*	BY4742 *atg12Δ::kanMX*	Euroscarf
Δ*atg14*	BY4742 *atg14Δ::kanMX*	Euroscarf
Δ*atg15*	BY4742 *atg15Δ::kanMX*	Euroscarf
Δ*atg16*	BY4742 *atg16Δ::kanMX*	Euroscarf
Δ*atg24*	BY4742 *atg24Δ::kanMX*	Euroscarf
Δ*atg27*	BY4742 *atg27Δ::kanMX*	Euroscarf
Δ*atg29*	BY4742 *atg29Δ::kanMX*	Euroscarf
Δ*atg31*	BY4742 *atg31Δ::kanMX*	Euroscarf
Δ*dal80*	BY4742 *dal80Δ::kanMX*	Euroscarf
Δ*gat1*	BY4742 *gat1Δ::HphNT1*	This study [a]
Δ*gcn4*	BY4742 *gcn4Δ::kanMX*	Euroscarf
Δ*gln3*	BY4742 *gln3Δ::HphNT1*	This study [a]
Δ*gzf3*	BY4742 *gzf3Δ::kanMX*	Euroscarf
Δ*msn2*	BY4742 *msn2Δ::kanMX*	Euroscarf
Δ*msn4*	BY4742 *msn4Δ::kanMX*	Euroscarf
Δ*pph21/22*	BY4742 *pph21Δ::URA3 pph22Δ::HphNT1*	This study [a]
Δ*ras2*	BY4742 *ras2Δ::kanMX*	Euroscarf
Δ*rim15*	BY4742 *rim15Δ::kanMX*	Euroscarf
Δ*sch9*	BY4742 *sch9Δ::URA3*	This study [a]
Δ*sir2*	BY4742 *sir2Δ::kanMX*	Euroscarf
Δ*snf1*	BY4742 *snf1Δ::natNT2*	This study [a]
Δ*snf4*	BY4742 *snf4Δ::kanMX*	Euroscarf
Δ*tor1*	BY4742 *tor1Δ::URA3*	This study [a]
Δ*tpd3*	BY4742 *tpd3Δ::kanMX*	Euroscarf
Δ*tpk1*	BY4742 *tpk1Δ::kanMX*	Euroscarf
Δ*tpk2*	BY4742 *tpk2Δ::kanMX*	Euroscarf
Δ*tpk3*	BY4742 *tpk3Δ::kanMX*	Euroscarf
Δ*ure2*	BY4742 *ure2Δ::kanMX*	Euroscarf
WT pATG8-yEGFP-ATG8	BY4742 pATG8::natNT2-pATG8-yEGFP	This study. [6]
Δ*gln3* pATG8-yEGFP-ATG8	BY4742 *gln3Δ::HphNT1* pATG8::natNT2-pATG8-yEGFP	This study
WT ATG7-6HA	BY4742 ATG7-6(HA)-natNT2	This study
WT ATG5-6HA	BY4742 ATG5-6(HA)-natNT2	This study
WT ATG29-6HA	BY4742 ATG29-6(HA)-natNT2	This study
WT *PHO8ΔN60*	BY4742 *pTN9(PHO8ΔN60)::URA3*	This study. [7]
WT (vector control)	BY4742 [pESC-HIS-6(HA)]	This study [b]
WT (GLN3 overexpression)	BY4742 [pESC-HIS-GLN3-6(HA)]	This study [b]
Δ*gln3* (vector control)	BY4742 [pESC-HIS-6(HA)]	This study [b]
Δ*gln3* (GLN3 overexpression)	BY4742 [pESC-HIS-GLN3-6(HA)]	This study [b]
WT (P_{MEP2}-*LacZ* fusion)	BY4742 [YCp*MEP2-LacZ*]	This study [c]

Δ*gln3* (P_{MEP2}-*LacZ* fusion)	BY4742 [YCp*MEP2-LacZ*]	This study [c]
WT (UPRE-*LacZ* fusion)	BY4742 [pMCZ-Y]	This study. [8]
WT Ub^{G76V}-GFP	BY4742 [pYES2-Ub-G76V-GFP]	This study. [9]

[a] To avoid clone-specific variations, at least four independent clones of the self-generated deletion mutant were verified (for primers. see Supplementary Table 4)

[b] This represents a modified version containing 6(HA) instead of FLAG tag (for primers, see Supplementary Table 5)

[c] The plasmid was a gift from Bruno André (Université Libre de Bruxelles. Belgium)

For details regarding strain construction refer to the 'Methods' section.

Supplementary Table 4 (related to all Figures): Primers used for yeast mutant generation

PCR template	Primer	Sequence (5'-3')
pUG27 (URA3)	atg1_pUG_f	TTCAAATCTCTTTTACAACACCAGACGAGAAATTAAGAAAcagctgaagcttcgtacgc
	atg1_pUG_r	GGTCATTTGTACTTAATAAGAAAACCATATTATGCATCACgcataggccactagtggatctg
	*atg1*Δ control primer	CGCTCGGCTCTGATTTCT
	URA3 control primer rev	TTGGCTAATCATGACCCC
pUG27 (URA3)	atg5_pUG_f	GGTTCTAGAAGAACGGAGATAGGAAACCTATGATGTAAGTcagctgaagcttcgtacgc
	atg5_pUG_r	GATATTTGAATGACACTTTTAAATGCGTATATAACAGCTCgcataggccactagtggatctg
	*atg5*Δ control primer	CGGCGAAGGGTATCTTCAT
	URA3 control primer rev	TTGGCTAATCATGACCCC
pUG27 (URA3)	atg7_pUG_f	TTCATTATATTTCAACAAATATAAGATAATCAAGAATAAAcagctgaagcttcgtacgc
	atg7_pUG_r	TGGCACCACAATATGTACCAATGCTATTATATGCAAAATAgcataggccactagtggatctg
	*atg7*Δ control primer	TGTACCTCCTGAAGAGCATGA
	URA3 control primer rev	TTGGCTAATCATGACCCC
pFA6a–hphNT1	gln3_S1	AGAGAGACGAGAGAGAGCACAGGGCCCCCTTTTCCCCCACCAACAAACAAcgtacgctgcaggtcgac
	gln3_S2	CGTTCAGTAATTATTAACATAATAAGAATAATGATAATGATAATACGCGGatcgatgaattcgagctcg
	*gln3*Δ control primer	GAGGACGCGCAGTAAGATAAG
	pYM control primer rev	GTCGACCTGCAGCGTACG
pUG27 (URA3)	pph21_pUG_f	AAAGAGGGATATAAATTATCGCATAAAACAATAAACAAAAAGAAAAcagctgaagcttcgtacgc
	pph21_pUG_r	AGAAAAGTGAATATATATCTATATAGATGCATATATGTATACATACgcataggccactagtggatctg
	*pph21*Δ control primer	TTTCCGGATTACATTAGTTCGG
	URA3 control primer rev	TTGGCTAATCATGACCCC
pFA6a–hphNT1	pph22_S1	GAATTTTATATTATTGGCACTTCTGTATAACTGGCTTTCATTCGAAAAAAcgtacgctgcaggtcgac
	pph22_S2	TATGTTGGAATGAAATAGCGTAGTAAGGATAAAGGTGTAATAGATATATAatcgatgaattcgagctcg

	*pph22*Δ control primer	TACATCCAGGAATAGAGTCCAC
	pYM control primer rev	GTCGACCTGCAGCGTACG
pFA6a–hphNT1	gat1_S1	GCCCAGCCACATATATATAGGTGTGTGCCACTCC CGGCCCCGGTATTAGCcgtacgctgcaggtcgac
	gat1_S2	TGAAGCGGACATGGAAAGAAGCGAGTACTTTTTT TTTTTTGGGGGATCTAatcgatgaattcgagctcg
	*gat1*Δ control primer fw	CCGCTTTCCGATTTTAGCCG
	pYM control primer rev	GTCGACCTGCAGCGTACG
pFA6a–natNT2	snf1_S1	TTTTTTTTGTAACAAGTTTTGCTACACTCCCTTAA TAAAGTCAACcgtacgctgcaggtcgac
	snf1_S2	CATAAAAAAAAGGGAACTTCCATATCATTCTTTTA CGTTCCACCATCAatcgatgaattcgagctcg
	*snf1*Δ control primer fw	CGTGATGATGGGACTCGA
	pYM control primer rev	GTCGACCTGCAGCGTACG
pYM-17 (natNT2)	Atg5_S2	TATTTTCTGCGATATTTGAATGACACTTTTAAATG CGTATATAACAGCTCatcgatgaattcgagctcg
	Atg5_S3	ACATAACTCTTGTTCCTATAAAAGGCGGCGATAA AGCTTCCTCTGAGCTCcgtacgctgcaggtcgac
	ATG5 control primer fw	GTTATGGGCGATTCGTTGC
	pYM control primer rev	GTCGACCTGCAGCGTACG
pYM-17 (natNT2)	Atg7_S2	TTACGGAAAGTGGCACCACAATATGTACCAATGC TATTATATGCAAAATAatcgatgaattcgagctcg
	Atg7_S3	TAGGCAACGATGTTTTTGAATGGGAAGATGATGA ATCTGATGAGATTGCTcgtacgctgcaggtcgac
	ATG7 control primer fw	GGCATGGTAATAGAGATGAACAG
	pYM control primer rev	GTCGACCTGCAGCGTACG
pYM-17 (natNT2)	Atg29_S2	GCCGACAGTTGGTTTTTTGATTGTGCTTGTGAAA GATGTAAATCAatcgatgaattcgagctcg
	Atg29_S3	AGTAAATCTGCGTTGGAAGAAGCGCTAATGGAC AGATTGCAATTCcgtacgctgcaggtcgac
	pYM control primer fw	CGAGCTCGAATTCATCGAT
	ATG29 control primer rev	AGCTTAGCAAAATATCTAAATTTCC

Cassette for deletion via homologous recombination was generated via PCR using the depicted plasmid/template as described in Gueldener et al. [10] (pUG system) or Janke et al. [11] (pYM system). Deletion was controlled via PCR of the inserted marker.

Supplementary Table 5 (related to all Figures): Primers used for cloning and siRNA

CLONING

**Target**	**Name**	**Sequence**
Angelica keiskei koidzumi nuclear rDNA	ITS4	TCCTCCGCTTATTGATATGC
	ITS5	GGAAGTAAAAGTCGTAACAAGG
S. cerevisiae GLN3	GLN3_F (*NotI*)	ATCTgcggccgcATGCAAGACGACCCCGAAA ATTC
	GLN3_R (*ClaI*)	ATCTatcgatGTTATACCAAATTTTAACCAATC CATTCGTCAGC
6(HA) (pYM17)	*ClaI*_6HA_f	tagtATCGATttCCGGTTCTGCTGCTAGATAC
	*SacI*_6HA_r	agatGAGCTCCGTTCCACTTTTTAGCTAGAA GC

siRNA

**Target**	**Name**	**Sequence**
-	siCtrl	UAGCGACUAAACACAUCAA
2623 (human gene ID)	siGATA1-1#	GGACAGGCCACUACCUAUG
2623 (human gene ID)	siGATA1-2#	AAGAAGCGCCUGAUUGUCA
2623 (human gene ID)	siGATA1-3#	GACCUGCACUGCCUUCAUC
2624 (human gene ID)	siGATA2-1#	UCGAGGAGCUGUCAAAGUG
2624 (human gene ID)	siGATA2-2#	ACUACAAGCUGCACAAUGU
2624 (human gene ID)	siGATA2-3#	GCCCAGGCCUAGCUACUAU
2625 (human gene ID)	siGATA3-1#	AGAAAGAGUGCCUCAAGUA
2625 (human gene ID)	siGATA3-2#	GUACAGCUCCGGACUCUUC
2625 (human gene ID)	siGATA3-3#	CGGCAGGACGAGAAAGAGU
2626 (human gene ID)	siGATA4-1#	CCAAGAACCUGAAUAAAUC
2626 (human gene ID)	siGATA4-2#	GGACAUAAUCACUGCGUAA
2626 (human gene ID)	siGATA4-3#	CGAUAUGUUUGACGACUUC
140628 (human gene ID)	siGATA5-1#	GGGUGAGGGUCGUGAGUGU
140628 (human gene ID)	siGATA5-2#	ACACGGAAGCGGAAGCCAA
140628 (human gene ID)	siGATA5-3#	GAAAGCAUCCAGACACGGA
2627 (human gene ID)	siGATA6-1#	GAACAGCGAGCUCAAGUAU
2627 (human gene ID)	siGATA6-2#	CAAGAUGGGCUCUACAUAG
2627 (human gene ID)	siGATA6-3#	GCAGAAACGCCGAGGGUGA
7227 (human gene ID)	siTRPS1-1#	GGACAAAUAUGACUUCACA
7227 (human gene ID)	siTRPS1-2#	CCACAGAUCUGAUUAAGCA
7227 (human gene ID)	siTRPS1-3#	CAACUCAUCCACCGAAUUA
2623 (human gene ID)	siATG5-1#	CAUCUGAGCUACCCGGAUA
2623 (human gene ID)	siATG5-2#	GACAAGAAGACAUUAGUGA
2623 (human gene ID)	siATG5-3#	CAAUUGGUUUGCUAUUUGA

Supplementary Table 6 (related to Figure 5): HPLC elution gradient applied for measurement of yeast metabolomics

Time (min)	Eluent B (%)	Pump flow (µL * min^{-1})
0	100	350
7	100	350
11	98	350
12	91	300
16	91	300
18	75	250
19	50	200
32	30	200
34	100	200
36	100	300
37	100	350
39	100	350

The table indicates the percentage of eluent B and the pumpflow (µL min^{-1}) during the course of elution (in min.). Compositions of eluent A and B are described in the corresponding 'Methods' section.

Supplementary References

1. Carmona-Gutiérrez, D. *et al.* The propeptide of yeast cathepsin D inhibits programmed necrosis. *Cell Death Dis.* **2**, e161 (2011).

2. Carmona-Gutierrez, D. *et al.* Guidelines and recommendations on yeast cell death nomenclature. *Microb. Cell* **5**, 4–31 (2018).

3. Rice-Evans, C. A. & Miller, N. J. Antioxidant activities of flavonoids as bioactive components of food. *Biochem. Soc. Trans.* **24**, 790–795 (1996).

4. Liu, C., Apodaca, J., Davis, L. E. & Rao, H. Proteasome inhibition in wild-type yeast Saccharomyces cerevisiae cells. *BioTechniques* **42**, 158, 160, 162 (2007).

5. Crozier, A., Jaganath, I. B. & Clifford, M. N. Dietary phenolics: chemistry, bioavailability and effects on health. *Nat. Prod. Rep.* **26**, 1001–1043 (2009).

6. Eisenberg, T. *et al.* Nucleocytosolic Depletion of the Energy Metabolite Acetyl-Coenzyme A Stimulates Autophagy and Prolongs Lifespan. *Cell Metab.* **19**, 431–444 (2014).

7. Noda, T. & Klionsky, D. J. The quantitative Pho8Delta60 assay of nonspecific autophagy. *Methods Enzymol.* **451**, 33–42 (2008).

8. Kawahara, T., Yanagi, H., Yura, T. & Mori, K. Endoplasmic reticulum stress-induced mRNA splicing permits synthesis of transcription factor Hac1p/Ern4p that activates the unfolded protein response. *Mol. Biol. Cell* **8**, 1845–1862 (1997).

9. Heessen, S., Dantuma, N. P., Tessarz, P., Jellne, M. & Masucci, M. G. Inhibition of ubiquitin/proteasome-dependent proteolysis in Saccharomyces cerevisiae by a Gly-Ala repeat. *FEBS Lett.* **555**, 397–404 (2003).

10. Gueldener, U., Heinisch, J., Koehler, G. J., Voss, D. & Hegemann, J. H. A second set of loxP marker cassettes for Cre-mediated multiple gene knockouts in budding yeast. *Nucleic Acids Res.* **30**, e23 (2002).

11. Janke, C. *et al.* A versatile toolbox for PCR-based tagging of yeast genes: new fluorescent proteins, more markers and promoter substitution cassettes. *Yeast Chichester Engl.* **21**, 947–962 (2004).
