# Supplementary material for: The flavonoid 4,4′-dimethoxychalcone promotes autophagy-dependent longevity across species
Source: Nat Commun. 2019 Feb 19;10:651. doi: 10.1038/s41467-019-08555-w (PMC6381180; doi:10.1038/s41467-019-08555-w)
Supplement: Supplementary file 3 — Description of Additional Supplementary Files [file 41467_2019_8555_MOESM3_ESM.docx]

**Description of Additional Supplementary Files**

**File Name:** Supplementary Data 1

**Description:** Yeast metabolome and pathway enrichment and topology analysis.

**File Name:** Supplementary Data 2

**Description:** Yeast proteome and GO term enrichment analysis.

**File Name:** Supplementary Data 3

**Description:** Mouse heart metabolome and pathway enrichment and topology analysis.

**File Name:** Supplementary Data 4

**Description:** Mouse liver metabolome and pathway enrichment and topology analysis.
