# Supplementary material for: The flavonoid 4,4′-dimethoxychalcone promotes autophagy-dependent longevity across species
Source: Nat Commun. 2019 Feb 19;10:651. doi: 10.1038/s41467-019-08555-w (PMC6381180; doi:10.1038/s41467-019-08555-w)
Supplement: Supplementary file 9 — Reporting Summary [file 41467_2019_8555_MOESM9_ESM.pdf]

nature research

Corresponding author(s): Frank Madeo, Guido Kroemer

☐ Initial submission ☐ Revised version ☒ Final submission

Life Sciences Reporting Summary

Nature Research wishes to improve the reproducibility of the work that we publish. This form is intended for publication with all accepted life science papers and provides structure for consistency and transparency in reporting. Every life science submission will use this form; some list items might not apply to an individual manuscript, but all fields must be completed for clarity.

For further information on the points included in this form, see Reporting Life Sciences Research. For further information on Nature Research policies, including our data availability policy, see Authors & Referees and the Editorial Policy Checklist.

Experimental design

1. Sample size

Describe how sample size was determined.	Sample sizes were chosen according to previous publications in the field. For animal studies, we adhered to the 3R principle.

2. Data exclusions

Describe any data exclusions.	No data was excluded.

3. Replication

Describe whether the experimental findings were reliably reproduced.	All experimental findings were reliably reproduced. Note that due to the rather high biological variance associated with ageing experiments, all single replicates of survival assays are shown.

4. Randomization

Describe how samples/organisms/participants were allocated into experimental groups.	Samples were allocated randomly to experimental groups.

5. Blinding

Describe whether the investigators were blinded to group allocation during data collection and/or analysis.	Investigators were not blinded during data collection and analysis.

Note: all studies involving animals and/or human research participants must disclose whether blinding and randomization were used.

6. Statistical parameters

For all figures and tables that use statistical methods, confirm that the following items are present in relevant figure legends (or in the Methods section if additional space is needed).

n/a	Confirmed	
☐	☒	The exact sample size (*n*) for each experimental group/condition, given as a discrete number and unit of measurement (animals, litters, cultures, etc.)
☐	☒	A description of how samples were collected, noting whether measurements were taken from distinct samples or whether the same sample was measured repeatedly
☐	☒	A statement indicating how many times each experiment was replicated
☐	☒	The statistical test(s) used and whether they are one- or two-sided (note: only common tests should be described solely by name; more complex techniques should be described in the Methods section)
☐	☒	A description of any assumptions or corrections, such as an adjustment for multiple comparisons
☐	☒	The test results (e.g. *P* values) given as exact values whenever possible and with confidence intervals noted
☐	☒	A clear description of statistics including central tendency (e.g. median, mean) and variation (e.g. standard deviation, interquartile range)
☐	☒	Clearly defined error bars

See the web collection on statistics for biologists for further resources and guidance.

Software

Policy information about availability of computer code

7. Software

Describe the software used to analyze the data in this study.	Origin Pro 8, Graphpad Prism 6, Microsoft Excel 2013

For manuscripts utilizing custom algorithms or software that are central to the paper but not yet described in the published literature, software must be made available to editors and reviewers upon request. We strongly encourage code deposition in a community repository (e.g. GitHub). *Nature Methods* guidance for providing algorithms and software for publication provides further information on this topic.

Materials and reagents

Policy information about availability of materials

8. Materials availability

Indicate whether there are restrictions on availability of unique materials or if these materials are only available for distribution by a for-profit company.	No restriction.

9. Antibodies

Describe the antibodies used and how they were validated for use in the system under study (i.e. assay and species).

All the antibodies used are commercially available and have been validated as indicated on the manufacturer's website and/or in previous publications. Further validation was achieved by employing negative/positive controls in each experiment. The following information is described for each antibody used: Name/ Raised in/ Dilution/Source/Catalogue number/Application:

- GFP/Mouse/1:5000/Roche/#1814460/in vivo (yeast)

- LC3B/Rabbit/1:1,000/Cell Signaling/#2775/in vitro (human cells) and in vivo (mice)

- SQSTM1/Mouse/1:10,000/Abnova/clone 2C11, #H00008878-M01/in vitro (human cells)

- GAPDH/Rabbit/1:10,000/Cell Signaling/clone D16H11, #5174/in vitro (human cells) and in vivo (mice)

- GATA1/Rabbit/1:500/Thermo Fisher Scientific #PA1099X/in vitro (human cells)

- GATA2/Rabbit/1:100/Thermo Fisher Scientific #710242/in vitro (human cells)

- GATA3/Rabbit/1:1,000/Cell Signalling Technology #5852/in vitro (human cells)

- GATA4/Rabbit/1:1,000/Cell Signalling Technology #36966/in vitro (human cells)

- GATA5/Goat/1:200/Thermo Fisher Scientific # PA547262/in vitro (human cells)

- GATA6/Rabbit/1:1,000/Cell Signalling Technology #5851/in vitro (human cells)

- TRPS1/Rabbit/1:2,000/Abcam # ab48820/in vitro (human cells)

- Atg5/Rabbit/1:1,000/Cell Signalling Technology #12994/in vitro (human cells)

- Mouse-IgG-HRP/Rabbit/1:10,000/Sigma/#F-9137/in vivo (yeast)

- Rabbit-IgG-HRP/Goat/1:10,000/Sigma/A0545/in vivo (yeast)

- Rabbit-IgG-Alexa Fluor®488/Goat/1:500/Invitrogen/#A11034/Drosophila brains

- Rabbit-IgG-HRP/Goat/1:5,000/Thermo Scientific/#31460/in vitro (human cells) and in vivo (mice)

- Mouse-IgG-HRP/Goat/1:5,000/Thermo Scientific/#31430/in vitro (human cells) and in vivo (mice)

The following antibodies were gifts from other researchers. The information described below for each antibody used: Name(Epitope)/Raised in/Dilution/ Source/doi of the original paper/Application:

- GAPDH/Rabbit/1:40,000/gift from Guenther Daum/doi: 10.1016/ j.bbalip.2009.01.015/in vivo (yeast)

- Ref(2)P/Rabbit/1:8,000/gift from Gabor Juhasz/doi: 10.1371/ journal.pone.0044214/Drosophila brains

10. Eukaryotic cell lines

a. State the source of each eukaryotic cell line used.	- U2OS (human osteosarcoma cell line was purchased from ATCC [HTB-96]; GFP-LC3 U2OS cells were generated by transfection of U2OS cells with pEGFP-LC3 plasmid and were maintained by selection with Neomycin).<br><br>- HCT116 (human colorectal carcinoma cell line was purchased from ATCC [CCL-247] GFP-LC3 HCT116 cells were generated by transfection with lentiviral GFP-LC3 construct (17-10193, Millipore).<br><br>- HepG2 (human hepatocellular carcinoma cell line was purchased from ATCC [HB8065]).
b. Describe the method of cell line authentication used.	Cell lines were not authenticated.
c. Report whether the cell lines were tested for mycoplasma contamination.	Cell lines have been tested negative for mycoplasma contamination.
d. If any of the cell lines used are listed in the database of commonly misidentified cell lines maintained by ICLAC, provide a scientific rationale for their use.	None of the cells used for this study were listed in the database of commonly misidentified cell lines that is maintained by ICLAC.

Animals and human research participants

Policy information about studies involving animals; when reporting animal research, follow the ARRIVE guidelines

11. Description of research animals

Provide details on animals and/or animal-derived materials used in the study.	Species: Mus musculus<br>Strains: C57Bl/6j.<br>Gender: male.<br>Age: The animals used were 6 weeks (autophagy), 3 months (prolonged ischemia), and 12 months (test feeding) old, respectively.

Policy information about studies involving human research participants

12. Description of human research participants

Describe the covariate-relevant population characteristics of the human research participants.	Study did not involve human research participants.
